# Chitosan and Its Derivatives as Highly Efficient Polymer Ligands

**DOI:** 10.3390/molecules21030330

**Published:** 2016-03-11

**Authors:** Alexander Pestov, Svetlana Bratskaya

**Affiliations:** 1 I.Ya. Postovsky Institute of Organic Synthesis, Ural Branch of Russian Academy of Sciences, Yekaterinburg 620990, Russia; pestov@ios.uran.ru; 2 Institute of Chemistry, Far East Branch of Russian Academy of Sciences, Vladivostok 690022, Russia

**Keywords:** chitosan, chitosan derivatives, chemical modification, metal ions, sorption, complexation, selectivity, ion binding mechanism

## Abstract

The polyfunctional nature of chitosan enables its application as a polymer ligand not only for the recovery, separation, and concentration of metal ions, but for the fabrication of a wide spectrum of functional materials. Although unmodified chitosan itself is the unique cationic polysaccharide with very good complexing properties toward numerous metal ions, its sorption capacity and selectivity can be sufficiently increased and turned via chemical modification to meet requirements of the specific applications. In this review, which covers results of the last decade, we demonstrate how different strategies of chitosan chemical modification effect metal ions binding by O-, N-, S-, and P-containing chitosan derivatives, and which mechanisms are involved in binding of metal cation and anions by chitosan derivatives.

## 1. Introduction

The good metal binding properties of chitosan have promoted a steady growth of interest in experimental and theoretical studies of the polymer and its derivatives’ interactions with metal ions [1,2,3] and the development of simple and economically sound methods for the synthesis of derivatives with increased sorption capacity and selectivity [1,2,4,5,6,7]. Such studies have dramatically extended the fields of application of chitosan as a polymer ligand. If decades ago the main interest in chitosan complexing properties was concerned with the prospects of its application to remove toxic and valuable metal ions [1,2], at present, formation of complex compounds with chitosan and its derivatives with metal ions is considered as an important stage in the fabrication of novel materials for optics [8], catalysis [9,10] and sorption [3]. Understanding of the metal binding mechanisms and estimation of complex stability are required to predict the antibacterial [11], antifouling [12], and antitumor [13] activity of metal-chitosan complexes and to simulate their behavior in biological media in the presence of other polymers and low-molecular weight ligands [14].

Undoubtedly, chitosan is one of the most efficient natural polymer ligands binding a broad range of metal ions, except those of alkali and alkali-earth metals, which do not have free d- and f-orbitals [1]. Different aspects of chitosan interactions with metal ions are described in quite a number of literature reviews, including those of the last decade [1,2,6,15,16] and demonstrate (Table 1) that the sorption capacity of chitosan varies significantly in dependence on sorption conditions as well as chitosan form, source, physical characteristics, and crosslinking method [17]. Gerente *et al.* published a comprehensive review on the parameters controlling the adsorption of metal ions on chitosan of various physical types in batch and column application that allows prediction of the material performance at large scale [15].

In order to increase the native polymer affinity toward specific metal ions and to change the selectivity and efficient sorption pH range, chemical modification with introduction of functional groups increasing the chitosan affinity to specific metal ions and ion-templating method have been applied rather extensively. Ion-templating is based on the formation in the chitosan matrix of cavities of specific sizes, which perfectly fit to the size of ions to be removed. In spite of the fact that numerous reviews devoted to the biosorption of metal ions on chitosan-based materials have been published [1,2,6,15,16], little attention has been paid to chitosan functional derivatives themselves [6,7]. Presentation of a very wide range of chitosan functional derivatives in the available literature was predominantly of descriptive character because of the limited number of derivatives under examination in each individual work and insufficient attention was paid to the effect of synthesis conditions on the structures of the derivatives produced. 

It is important to emphasize that despite numerous reports on the applicability of chitosan for recovery of metal ions from wastewaters, one can hardly expect utilization of large volumes of chitosan-based sorbents for liquid waste treatment at an industrial scale due to the substantially higher price of chitosan in comparison with that of synthetic polymers and ion-exchange resins. However, high affinity of chitosan and high selectivity of its chemically modified derivatives to many metal ions promote the interest to application of these materials to pre-concentrate metal ions from wastewaters, natural waters, and biological and geological samples for subsequent analytical determination using atomic absorption spectrometry (ААS) [33,34,35]. Another field where chitosan derivatives with high affinity to metal ions find an application is green synthesis of metal nanoparticles, which can be used as catalysts [9] and optically active [36] or antibacterial [37] materials.

The main objective of the present review consists in the demonstration of which chitosan chemical modification strategies result in the formation of derivatives capable of the most efficient interactions with metal ions. In view of this, the review is devoted exclusively to the derivatives, whose introduced functional groups are formed on the basis of O-, N-, S-, and P-containing substituents. In particular, special attention was also paid to crosslinked chitosan derivatives, since chitosan crosslinking shall be also considered as a chemical modification, whereas graft copolymers were not discussed here, since their properties are mainly affected by the nature of the grafted polymer.

The relative efficiency of chitosan derivatives as polymer ligands for metal ions was estimated from the data on their sorption capacity toward metal ions, which was manifested in the most clearly expressed group selectivity and high values of the sorption capacity. This type of interaction correlates to the stability of metal complexes with chitosan derivatives that depends on the structure of the formed chelate compound and is determined by the degree and character of substitution by functional fragments.

Based on the chemical structure, functional chitosan derivatives can be divided into two main types: linker-containing derivatives and derivatives without linkers, if linkers are defined as additional structural fragments between the introduced functional substituent and the polymer backbone. Analysis of chitosan derivatives in this review was performed in accordance with this classification.

## 2. Chitosan Derivatives without Linkers

### 2.1. Main Synthesis Methods

#### 2.1.1. O-Containing Chitosan Derivatives

**Figure 1:**
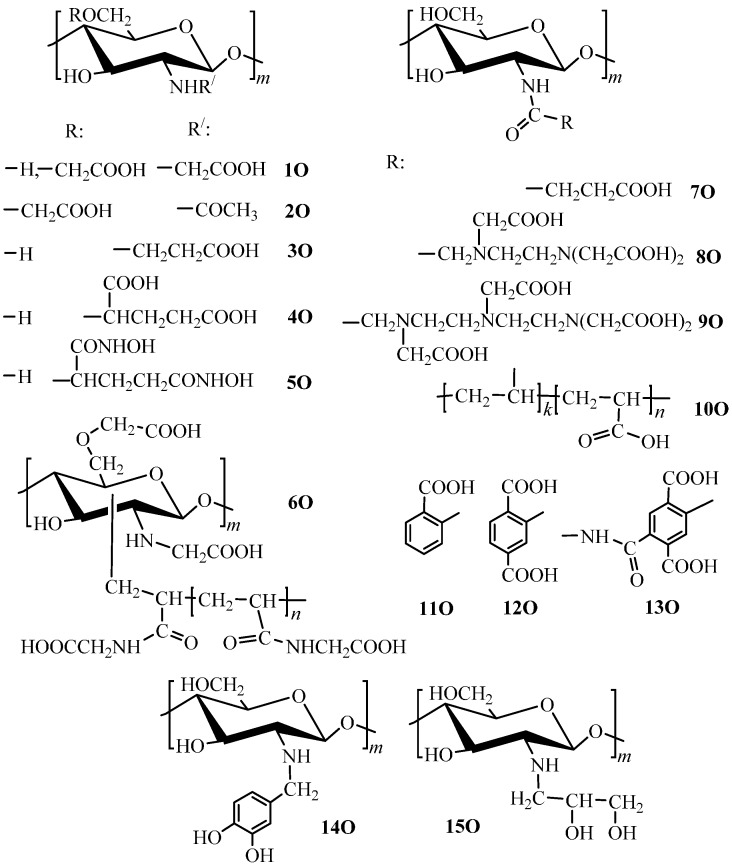
Structures of O-containing derivatives of chitosan without linkers: **1O**—*N*,*O*-carboxymethyl chitosan; **2O**—carboxymethyl chitin; **3O**—*N*-(2-carboxy)ethyl chitosan; **4O**—chitosan α-ketoglutaric acid (*N*-(1,3-dicarboxy)propyl chitosan); **5O**—hydroxamated chitosan α-ketoglutaric acid (*N*-(1,3-di(*N′*-hydroxy)carbamoyl)propyl chitosan); **6O**—carboxymethyl chitosan graft copolymerization of *N*-acryloylglycine; **7O**—*N*-succinyl chitosan; **8O**—EDTA-modified cross-linked chitosan; **9O**—DTPA-modified cross-linked chitosan; **10O**—chitosan granules functionalized with poly(acrylic acid); **11O**—chitosan-phthalate; **12O**—trimellitic anhydride-crosslinked chitosan; **13O**—chitosan-1,2,4,5-benzenetetracarboxylilic acid; **14O**—*N*-(3,4-dihydroxybenzyl) chitosan; **15O**—2,3-dihydroxypropyl chitosan.

Enhancement of metal binding properties of O-containing chitosan derivatives is provided the most efficiently through introduction of hydroxyl and carboxyl groups via suitable substitution and addition reactions. Presently, synthesis of carboxyalkyl chitosan derivatives is the most thoroughly developed polymer transformation methodology that is based on methods of carboxymethyl chitosan synthesis known since the 1980s [38]. The first method consists in the substitution reaction of chloroacetic acid with chitosan in alkaline medium [39,40,41] and serves today as a basis for manufacturing commercial carboxymethyl chitosan (**1О**, Figure 1) with the degree of substitution equal to 1. The second method [42,43,44] employs addition of chitosan to glyoxylic acid with subsequent reduction by borohydrides [45] that results in a product with a high degree of selective *N*-substitution (up to 2) [38]. Synthesis of the next homolog—carboxyethyl chitosan (**3O**, Figure 1)—is feasible using 3-halopropionic acids [46] or acrylic acid under “synthesis-in-gel” conditions, which allows efficient production of *N*-(2-carboxy)ethyl chitosan with a degree of substitution up to 1.6 [47]. The glutaric acid residue is introduced through reductive alkylation (**4О**, Figure 1) [48] followed by additional transformation of carboxyl groups into hydroxamated derivatives (**5О**, Figure 1).

Another method of carboxyl group introduction into the chitosan macromolecule is *N*-acylation. For this purpose, an anhydride reagent [18,22,24,49,50] or carbodiimide coupling reagent [27] is used. As a result, derivatives with degrees of acylation of 0.4–1 (**7О**–**13О**, Figure 1) are obtained. In comparison to carboxyalkylation, the acylation method enables one to introduce more carboxyl groups per glucosamine unit, but such derivatives can easily destroyed during the final application due to lower stability of the amide group in alkaline or acidic media.

Functionalization by carboxyl groups is also possible using the graft copolymerization of *N*-acryloylglycine onto carboxymethyl chitosan (**6O**, Figure 1) with a 157% grafting percentage [51].

In order to transform carboxylate derivatives into insoluble sorption materials, standard (glutaraldehyde [40,52,53], epichlorohydrin [22,50] or ethylene glycol diglycidyl ether [27]) as well as less common *N*,*N*′-methylenebis(acrylamide) [42], hexamethylene diisocyanate [24], or urea-formaldehyde snake-cage resin [43] crosslinking agents are used. The method of crosslinking carboxyalkyl derivatives by electron beam without changes to the polymer molecular weight is known as well [44,54]. In some chitosan modification cases via acetylation or addition in gel reactions, subsequent crosslinking is not required, since the reagent provides both functionalization and crosslinking of macromolecules [18,49,55] due to formation of amide groups.

As compared to carboxylation, chitosan modification using significantly weaker OH-acids is rather poorly developed. For this purpose, substitution reactions (**15O**, Figure 1) [56,57,58] and reductive alkylation (**14O**, Figure 1) [59] are used. A common disadvantage of these modification methods consists in the low degree of substitution obtained, not exceeding 0.3.

#### 2.1.2. N-Containing Chitosan Derivatives

Functionalization of chitosan by N-containing groups is carried out by introduction of an aliphatic amine, aromatic amine or heterocyclic fragment residue via suitable addition or substitution reactions of the already formed N-containing functional group.

**Figure 2:**
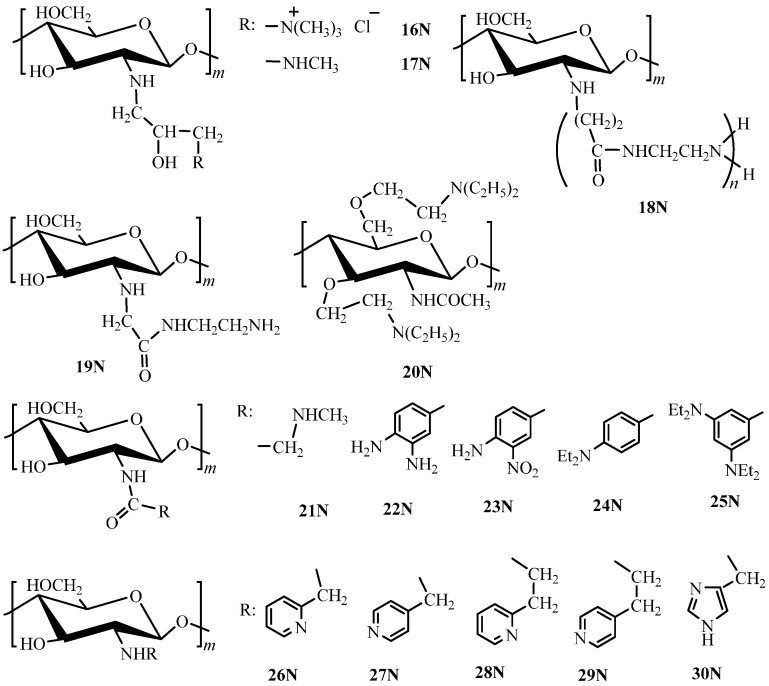
Structure of N-containing derivatives of chitosan without linkers: **16N**—*N*-(3-trimethylammonium-2-hydroxy)propyl chitosan; **17N**—*N*-(2-hydroxy-3-methylamino)propyl chitosan; **18N**—chitosan functionalized by amino-terminated hyperbranched polyamidoamine polymers; **19N**—aminated chitosan beads (*N′*-(2-aminoethyl)carbamoylmethyl chitosan); **20N**—*O*-(2-dimethylamino)ethyl chitin; **21N**—*N′*-methylglycyl chitosan; **22N**—chitosan resin functionalized with 3,4-diaminobenzoic acid (*N*-(3,4-diaminobenzoyl) chitosan); **23N**—chitosan resin functionalized with a 3-nitro-4-aminobenzoic acid moiety (*N*-(3-nitro-4-aminobenzoyl) chitosan); **24N**—*N*-(4-ethylaminobenzoyl) chitosan; **25N**—*N*-3,5-di(ethylaminobenzoyl) chitosan; **26N**—*N*-(2-pyridyl)methyl chitosan; **27N**—*N*-(4-pyridyl)methyl chitosan; **28N**—*N*-2-(2-pyridyl)ethyl chitosan; **29N**—*N*-2-(4-pyridyl)ethyl chitosan; **30N**—*N*-(4-imidazolyl)methyl chitosan.

The strictly formal amine alkylation is carried out by the standard method of nucleophilic substitution or through pendant modification using linkers. Using the first approach *O*-(2-dimethylamino)ethyl chitin (**20N**, Figure 2), *N*-(3-trimethylammonium-2-hydroxy)propyl chitosan (**16N**, Figure 2), and *N*-(2-hydroxy-3-methylamino)propyl chitosan (**17N**, Figure 2) with degrees of substitution of 1.85, 0.6, and 0.2, respectively, were produced through chitosan treatment by 2-chloroethyldimethylamine [60] or glycidyl trimethylammonium chloride [61] and treatment of *N*-(3-chloro-2-hydroxy)propyl chitosan by methylamine [62], respectively. 

Aminoarylation is carried out using derivatives of aromatic amino acids, which enables one to consider this modification approach as an example of functionalization by aliphatic aminoacids, including standard acylation reactions (**22N**–**25N**, Figure 2) [63,64,65,66]. One should mention that to form the amide group, a carbodiimide coupling reagent and pre-crosslinked chitosan are often used [63,66,67], while the less often used method here is more accessible water distillation [64,65]. In the latter case, the authors did not perform extra crosslinking. Chitosan functionalization by aliphatic aminoacids is also carried out using acylation reactions (**21N**, Figure 2) [68,69,70] that yield amino-terminated hyperbranched polyamidoamine polymers (**18N**, **19N**, Figure 2) [69,70].

Synthesis of chitosan derivatives containing heterocyclic fragments has been known since the 1990s. For example, to introduce a pyridine ring into the chitosan molecule, one of the most widely used methods of chitosan functionalization (reductive alkylation) is applied. Here, one obtains the Schiff base from 2- or 4-pyridinecarboxaldehyde (2-pyridinecarboxaldehyde, 4-pyridinecarboxaldehyde) [71,72,73,74] or, with subsequent treatment by borohydrides, *N*-(2-pyridyl)methyl chitosan (**26N**, Figure 2) or *N*-(4-pyridyl)methyl chitosan (**27N**, Figure 2) [73,75,76,77]. These derivatives with a degree of substitution 0.85–0.9 are further crosslinked by epichlorohydrin to obtain the sorption materials. To synthesize the next homologs *N*-2-(2-pyridyl)- and *N*-2-(4-pyridyl)ethyl chitosan (**28N**, **29N**, Figure 2) with a degree of substitution up to 1.0, the reaction of chitosan addition to 2- or 4-vinylpyridine can be used [78,79]. The essential feature of the method of synthesis of these derivatives consists in using the gel-synthesis effect, *i.e.*, using of the optimal concentration of the polymer gel (15%) providing the maximal degree of substitution. In general, this synthesis method is distinguished by simplicity of a single stage modification without usage of organic solvents and unstable and toxic hydride reducers. Crosslinking of the **28N** derivative (Figure 2) is provided by glutaraldehyde [80] or epichlorohydrin [78].

Another heterocyclic fragment used in chitosan modification is imidazole. This functionalization proceeds via substitution reaction of a chloromethyl derivative [81] or 4(5)-imidazomethanol [82] with chitosan or reductive alkylation using imidazole-4- or imidazole-2-carbaldehyde [83]. The final product here is *N*-(4-imidazolyl)methyl chitosan (**30N**, Figure 2) with degrees of substitution equal to 0.75, 0.35, and 0.3, respectively. To use **30N** derivative as a sorbent, crosslinking by oxirane derivatives was performed [82].

#### 2.1.3. S-Containing Chitosan Derivatives

**Figure 3:**
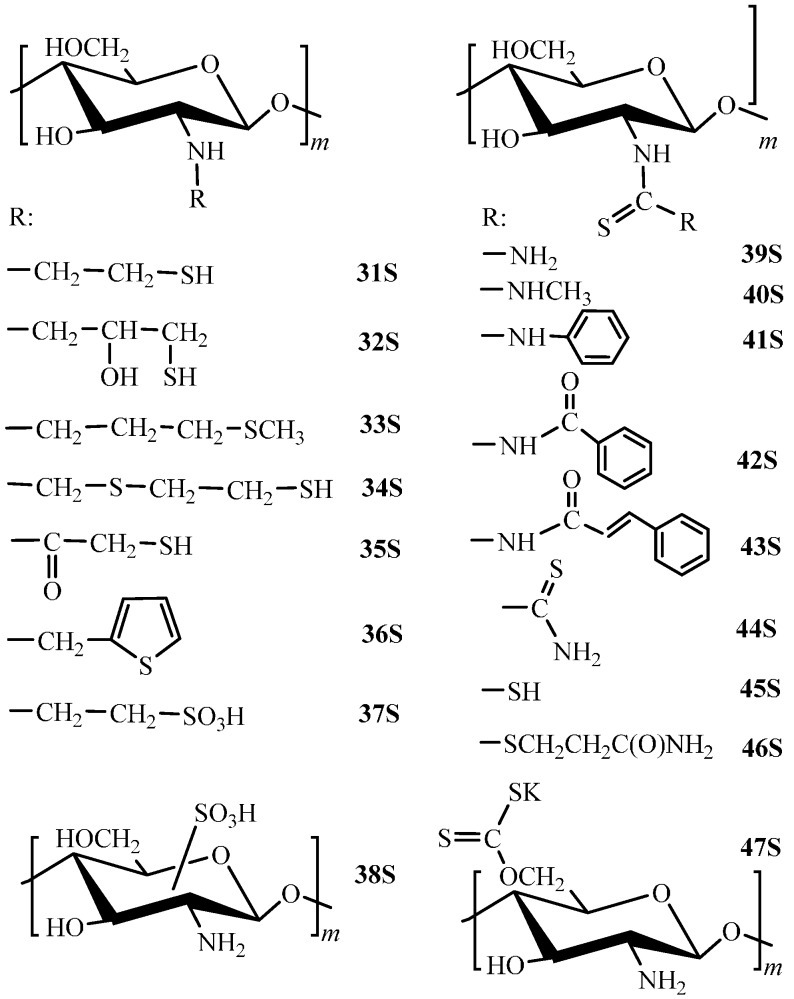
Structure of S-containing derivatives of chitosan without linkers: **31S**—chitosan ethylenesulfide (*N*-2-mercaptoethylchitosan); **32S**—*N*-(2-hydroxy-3-mercapto)propyl chitosan; **33S**—*N*-3-(methylthio)propyl chitosan; **34S**—chitosan derivative of 1,2-ethanedithiol (*N*-(4-mercapto-2-thiobutyl) chitosan); **35S**—*N*,*O*-mercaptoacetyl chitosan; **36S**—*N*-(2-thienylmethyl) chitosan; **37S**—*N*-(2-sulfo)ethyl chitosan; **38S**—chitosan sulfate; **39S**—*N*-thiocarbamoyl chitosan; **40S**—*N*-(methylthiocarbamoyl) chitosan; **41S**—*N*-(phenylthiocarbamoyl) chitosan; **42S**—chitosan benzoyl thiourea derivative (*N*-(benzoylthiocarbamoyl) chitosan); **43S**—chitosan cinnamoyl thiourea derivative (*N*-(cinnamoylthiocarbamoyl) chitosan); **44S**—dithiooxamide chitosan derivative; **45S**—chitosan dithiocarbamate; **46S**—dithiocarbamate modified chitosan (*S*-(2-carbamoylethyl) dithiocarbamate chitosan); **47S**—chitosan xanthate.

To obtain chitosan derivatives with sulfur-containing functional groups (mercapto- and thiocarbonyl derivatives), acylation, addition, and substitution reactions are used. Direct chitosan thiolation is provided through the reaction of addition to ethylene sulfide [84], acylation by thioglycolic acid [62,85], and formation of the aminothiol group of 1,2-ethanedithiol with formaldehyde as both crosslinking and linker-forming agent [86] (Figure 3). The final products here are *N*-2-mercaptoethyl chitosan (**31S**) and *N*,*O*-mercaptoacetyl chitosan (**35S**) with the degrees of substitution equal to 1.94 and 1.95, respectively, and *N*-(4-mercapto-2-thiobutyl) chitosan (**34S**) with the sulfur content equal to 2.54 mmol/g, which corresponds, supposedly, to the degree of substitution lower than 0.4. The standard method of mercaptan synthesis using thiourea is also well-known in chitosan chemistry [87]. Here, an additional functionalization by the hydroxyl group with formation of *N*-(2-hydroxy-3-mercapto)propyl chitosan (**32S**) with a degree of substitution equal to 0.54 takes place. One should mention an erroneous false conclusion made in subsequent works using this method of modification stating that it yields a chitosan thiourea derivative [88,89]. In fact, the authors synthesized the derivative **32S**, whose structure and composition were not analyzed at all and, therefore, the compound was not correctly identified.

Functionalization of chitosan by alkyl sulfide and sulfur-containing arenes was described in the literature as well [75]. It was carried out using sequential reactions of imine formation from respective the aldehyde and chitosan and its reduction by sodium borohydride. In this way, *N*-3-(methylthio)propyl chitosan (**33S**, Figure 3) and *N*-(2-thienyl)methyl chitosan (**36S**, Figure 3) with the degree of substitution equal to 0.9 for both derivatives were synthesized, although the authors did not present any corroboration for their proposed composition and structure.

To obtain thiocarbonyl derivatives, direct modification via addition reactions to carbon disulphide [90,91,92], isothiocyanates [93,94,95], thiocyanates [96], thiourea, and dithiooxamide [97] was used (Figure 3). As a result, the substituted (**40S**–**43S**) and unsubstituted (**39S**) *N*-thiocarbamoyl- chitosans with a degree of substitution up to 0.9, dithiooxamide chitosan derivative (**44S**) with the degree of substitution not higher than 0.4, chitosan xanthate (**47S**), and substituted (**46S**) and unsubstituted (**45S**) chitosan dithiocarbamates with degrees of modification not higher than 0.6 were obtained.

Sulfo-containing derivatives are obtained through direct sulfonation of chitosan (**38S**) [98] or by the substitution reaction using 2-chloro- or 2-bromoethanesulfonates (**37S**, Figure 3) [99]. To ensure insolubility of S-containing derivatives in acidic media, standard crosslinking agents are used for chitosan and its derivatives: glutaraldehyde [30,31,99,100,101], formaldehyde [86], and epichlorohydrin [75]. The crosslinking fragment could be also formed immediately in the derivative synthesis process [62,84,90,93,96] that yields the insoluble sorption material in a single step.

#### 2.1.4. P-Containing Chitosan Derivatives

**Figure 4:**
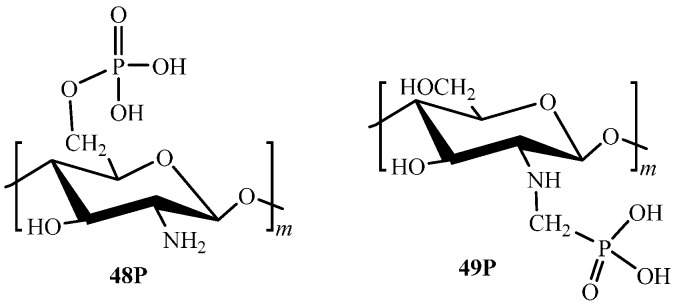
Structure of P-containing derivatives of chitosan without linkers: **48P**—chitosan O-phosphate; **49P**—*N*-methylenephosphonic acid chitosan.

Functionalization of chitosan by P-containing groups is reported rather extensively in the literature [102], whereas only two derivatives were used as sorption materials. Chitosan O-phosphate (**48P**, Figure 4) was obtained through the acylation reaction of polymer by phosphorus pentoxide in methanesulphonic acid [103] or phosphorus(V) oxychloride in DMF [104].

*N*-methylenephosphonic chitosan (**49Р**, Figure 4) is obtained using the Mannich reaction through condensation of chitosan, phosphorous acid, and formaldehyde [105,106]. Such methods of chitosan functionalization are efficient, since they allow the production of derivatives with high degrees of substitution up to 1.3 and 1.6, respectively. Derivatives crosslinking is performed using standard agents, for example, epichlorohydrin [104] or adipoyl chloride [103].

### 2.2. Sorption Properties 

#### 2.2.1. O-Containing Chitosan Derivatives

Introduction of carboxyl groups into chitosan is an efficient way of controlling the derivative selectivity and sorption capacity due to formation of stable chelate rings with transition metal ions, whose size is determined by the substituent structure. The most thoroughly studied derivatives of this type are carboxyalkyl chitosans [39,40,42,46,47,52,53] and derivatives containing fragments of ethylenediaminetetraacetic acid (EDTA) [22,49,107] and diethylenetriaminepentaacetic acid (DTPA) [49,107].

Twofold and larger increase of the sorption capacity of O-containing chitosan derivatives, as compared to that of unmodified polymer, was observed for a wide range of metal ions—Cu(II) [18,22,39,40,47,51,53,57], Ni(II) [22,39,49,53], Co(II) [22,49,53], Zn(II) [48,53]—see Table 2. Here, the sorption capacity values provided by different authors for derivatives of the same type vary over a significantly narrower range in comparison with that for the native polymer, which allows concluding that introduction of high-affinity substituents reduces the effect of the chitosan source and the characteristics on the sorption properties of the obtained materials (Table 2).

Introduction of carboxyl groups ensures the chitosan’s ability to sorb metal ions, for which the efficiency of binding by the unmodified polymer is negligibly small. For example, it was demonstrated that a gel of carboxymethyl chitosan (**1O**, Figure 1) with a degree of substitution equal to 1.78 crosslinked using gamma-radiation manifested the sorption capacity for Sr(II) ions higher than 1 mmol/g at рН 4–8 [42], while native chitosan does not sorb earth-alkali metal ions.

A substantial increase of the sorption capacity toward Pb(II) ions (it does not exceed 0.3 mmol Pb/g for the native chitosan [20,28]) was mentioned for *N*-succinyl chitosan (**7O**, Figure 1). For a series of derivatives with the degree of acylation up to 0.6, the maximal sorption of Pb(II) ions was equal to 1.68 mmol/g at рН 5.8 [53]. Comparable, albeit somewhat lower, sorption capacity values were reported for chitosan acylated by polyacrylic acid [27] and EDTA [22]. 

The possibility of chelate ring formation between chitosan derivatives and metal ions has a substantial effect on both capacity and selectivity of sorption. Indeed, carboxyl chitosan derivatives incapable of chelation (**2О**, **4О**, **5О**, **10О**–**13О**, Figure 1) manifest lower sorption capacity (Table 2) than those forming five- (**1О**, **6О**, **8О**, **9О**, Figure 1) and six-membered (**3О**, Figure 1) chelate rings. One should specially mention a substantial difference in the values of sorption capacity for carboxymethyl chitosan (**1О**, Figure 1) and carboxymethyl chitin (**2О**, Figure 1) [44,45,54,108], whereas a systematic comparison of their sorption properties is not possible because of different chemical structures of derivatives of the same type and not optimal sorption conditions.

Studies of sorption properties of chitosans modified with the most efficient complexing agents ethylenediaminetetraacetic acid (EDTA, **8O**, Figure 1) and diethylenetriaminepentaacetic acid (DTPA) (**9O**, Figure 1)) [22,49,107] demonstrated that the rows of selectivity of metal ions removal corresponded to the rows of stability of their chelates with respective low-molecular ligands (Ga(III)~In(III)~Fe(III) > Cu(II)~Mo(VI) > Ni(II) > V(IV)~Zn(II)~Co(II)~Al(III)~Mn(II)), which indicates to preservation of the chelating ability of the ligand upon its grafting to the polymer chain. Introduction of these substituents enables one not only to increase the sorption capacity, but also to significantly extend the pH range of efficient sorption of metal ions. Whereas the sorption of cationic forms of transition metals is maximal at pH 4–6 for the native chitosan [20] and at рН > 4 for carboxyalkyl chitosan and *N*-succinyl chitosan [39,42,52], chitosans acylated with EDTA and DTPA sorb efficiently ions of many transition metals at pH 1–2. Comparing the sorption capacity of **8O** and **9O** derivatives, Figure 1 (Table 2), one can see that the **8O** derivatives are characterized with higher values of the sorption capacity, although the constants of stability of the complex (for instance, that of Cu(II) ions with EDTA) are 3–4 orders of magnitude lower than those for DTPA. Most probably, this is related to different degrees of substitution, which is equal to 100% in case of acylation by EDTA fragments, while just 22% of chitosan amino groups were acylated by DTPA fragments due to steric hindrances [107].

The effect of the degree of substitution on the sorption properties of carboxylated chitosan derivatives was examined in a number of works [39,46,47,52,53,55]. For example, for *N*-succinyl chitosans (**7O**, Figure 1) with a degree of substitution from 0.15 to 0.6, it was found that the sorption capacity toward Pb(II) ions increased along with the increase of the DS up to 0.25, after which it remained virtually constant [53]. The nonlinear dependence of the sorption capacity on the degree of substitution was also established for carboxymethyl chitosans (**1O**, Figure 1) for sorption of Cu(II) [39] and Zn(II) ions [52]. The capacity value increased significantly in the DS ranges 0.62–1.06 [39] and 0.72–0.96 [52], respectively, and decreased noticeably at further increase of the derivatives DS that was related by the authors to participation of different types of groups (-COOH, -NH_2_, -OH) with different affinities in the chelates formation. The increase of the degree of substitution affects the sorption properties of the next homolog (carboxyethyl chitosan, **3O**, Figure 1) nonlinearly as well [47,55]: the maximal degree of modification provides neither the highest capacity nor the highest sorption selectivity with respect to Cu(II), Ni(II), Zn(II), and Co(II) ions.

A rather successful attempt to explain changes in how the degree of substitution affects metal binding properties of carboxyalkyl chitosans was made in [46] on the basis of the results of determination of complex formation constants using the potentiometric titration method. For instance, a dramatic decrease of stability constants (logβ) for complexes of carboxyethyl chitosan (**3O**, Figure 1) with Cu(II) ions from 11.6 to 6.41, when the degree of substitution increased from 0.92 to 1.61, was reported by the authors to be due to the transition from the bridge model of Cu(II) ions binding to the pendant model that corresponds the metal:ligand complex ratios 1:2 and 1:1, respectively. Subsequent EPR studies of copper complexes of the **3O** derivatives corroborated the suggested complex structures [47,55].

Despite the fact that the selectivity of carboxymethyl chitosan to Cu(II) ions is significantly higher in comparison to the unmodified polymer [40], the data shown in Table 2 confirm that a majority of O-containing chitosan derivatives sorb a number of metal cations rather efficiently. As shown in [14], the complex stability constants for metal ions and *N*,*O*-carboxymethyl chitosan differed by less than two orders of magnitude for ion pairs Ni(II)/Co(II), Ni(II)/Zn(II), and Co(II)/Mn(II) that allows assuming only a group selectivity of the derivative toward transition metal ions. Efficient separation of Ni(II) and Co(II) ions [49,107] and selective concentration of Ni(II) and Co(II) ions from solutions with high content of Al(III) ions [107] was demonstrated only for derivatives with EDTA and DTPA fragments.

In order to increase the selectivity of O-containing chitosan derivatives [52,53,109], the template synthesis method is applied, similarly to the native chitosan [110]. Use of Pb(II) ions allowed synthesis of the sorbent based on *N*-succinyl chitosan (**8O**) providing a significant increase of the selectivity of removal of Pb(II) ions from solutions containing Cd(II), Hg(II), and Zn(II) ions [53]. Templating carboxymethyl chitosan (**1O**, Figure 1) with Zn(II) ions ensured the increase of separation coefficients for Zn(II)/Cu(II) and Zn(II)/Pb(II) pairs by 68 and 8 times, respectively, despite some decrease of the material sorption capacity as a result of crosslinking [52].

In most cases, hydroxyl derivatives of chitosan (**14О**, **15О**, Figure 1) are characterized by lower sorption capacity values in comparison with carboxylated derivatives. This is expected because of weaker electron donor properties of the hydroxyl group [59]. At the same time, formation of less stable complexes with d-metals ions provides the possibility of application of such derivatives as substrates for metal-containing catalysts [71,72,111], in which a labile coordination sphere of the metal center is required. On the other hand, due to high affinity of a number of nonmetals to polyol compounds, hydroxyalkyl derivatives of chitosan (**15O**, Figure 1) are highly efficient sorbents for the removal of Ge(IV) ions [112]. Selective sorption of Ge(IV) on such materials in the presence of Te(IV), B(III), As(III), As(V), and Se(VI) ions is possible [56].

#### 2.2.2. N-Containing Chitosan Derivatives 

Introduction of aliphatic and aromatic amines into the chitosan structure is one of the most simple and efficient methods of increasing the number of electron-donor nitrogen atoms, which can serve as sorption sites for both cations and anions of metals depending on the binding conditions. As seen from Table 3, the most pronounced increase of sorption capacities of this type of derivatives, as compared to the unmodified chitosan, was found for Hg(II) ions [69,70,113,114] and anionic complexes of noble metals [87]. For a number of derivatives, a significant increase of the sorption capacity was observed for other metal ions as well.

The substituent structure has a substantial effect on the derivatives sorption properties. For example, a dramatic decrease of the sorption capacity of *N*-(4-ethylaminobenzoyl) chitosan (**24N**, Figure 2), as compared to that of *N*-(3,5-diethylaminobenzoyl) chitosan (**25N**, Figure 2), found in [65] was related by the authors to low efficiency of Cu(II) ions coordination of the substituent amino group in the *para*-position. Aminoarylation allows increasing the chitosan selectivity to As(V), Se(IV), and Se(VI) [63] ions. Introduction of the anthranilic acid residue ensures the possibility to concentrate a number of transitions metals ions and lanthanides for subsequent atomic absorption determination [67].

Introduction of *N*-heterocyclic fragments (pyridyl- and imidazolyl-containing substituents) capable of forming stable five- and six-membered chelate rings into the chitosan structure is also a popular method to increase the sorption capacity and selectivity of the native chitosan. For instance, a substantial increase of the sorption capacity of *N*-(2-pyridyl)methyl chitosan (**26N**, Figure 2) toward Cu(II) ions was related to formation of a five-membered chelate ring [76,124], which cannot be formed in the case of the 4-pyridyl derivative (**27N**, Figure 2), which displays almost threefold lower sorption capacity [115]. In spite of the fact that the stability constants of Cu(II) complexes with monomer ligands (2-(aminomethyl)pyridine and 2-(2'-aminoethyl)pyridine), forming five- and six-membered chelate rings, respectively, differ by four orders of magnitude [125], the sorption capacity of *N*-2-(2-pyridyl)ethyl chitosan appeared to be just insignificantly lower than that of *N*-(2-pyridyl)methyl chitosan. However, the capacity of 4-pyridyl derivative is lower than that of 2-pyridyl derivative in case of ethyl homologs as well [79]. 

Comparison of sorption properties of imidazolyl chitosan derivatives [82] with those of known pyridyl derivatives [76,78] revealed a number of important facts. First, the sorption capacity of the low-substituted *N*-(4-imidazolyl)methyl chitosan (**30N**, Figure 2) for Ag(I) ions is higher than that of high-substituted pyridylethyl derivatives (**28N**, Figure 2). Second, unlike pyridyl chitosan derivatives with the clearly expressed selectivity row Cu(II) > Ni(II) > Co(II), imidazolyl derivatives are characterized with rather high sorption capacity with respect to Co(II) and Ni(II) ions [82]—see Table 3. Higher affinity of the imidazolyl chitosan derivative to Co(II) ions, as compared to that of the native polymer, allowed its application for immobilization of catalytically active Co(II) complex with *N*,*N′*-ethylenebis(salicylimine) [74].

As in the case of amine chitosan derivatives, introduction of pyridyl and imidazolyl fragments substantially increases the chitosan sorption capacity toward anionic complexes of noble metals [75,78,82], which is related, first of all, to the increase of the quantity of nitrogen atoms in the macromolecule protonated in the acidic medium and, therefore, of the anion-exchange sites. The sorption capacity changes in the row Au(III) > Pd(II) > Pt(IV) that is typical for N-containing polymeric sorbents. Here, it is important to mention that the sorption capacity depends significantly not only on the degree of substitution, but also on the structure of the material formed in the crosslinking process. The decrease of the sorption capacity along with the increase of the crosslinking degree and the polymer matrix rigidity was demonstrated for imidazolyl chitosan derivatives [82].

#### 2.2.3. S-Containing Chitosan Derivatives

The targeted chemical modification by S-containing functional groups is widely used to improve sorption properties of chitosan toward ions of noble [30,31,87,89,96,100] and some transition [85,92,93,126] metals through formation of additional coordination centers and eliminating effects of pH and competing ions on sorption properties. Up to present, the sorption properties of chitosans modified by thiourea [30,96,97,101,127,128] and dithiooxamide [30,31,97] and of chitosan dithiocarbamate [90,91] and mercapto derivatives [62,87,88,89,95] have been investigated in detail.

Comparative data on the sorption properties of sulfur-containing derivatives toward ions of noble and transition metals (Table 4) demonstrate that, depending on the substituent type, synthesis method, and degree of substitution, the materials capacity and affinity vary in very broad ranges. The covalent grafting of the N-substituted thiourea directly to the polymer framework without using linkers (**40S** and **41S**, Figure 3) [93] yielded the derivatives characterized with very low sorption capacity (0.3 mmol/g for Pd(II) and Au(III) at the degree of substitution equal to 0.9), most probably, because of the uncontrolled degree of crosslinking by isocyanate.

The method of chitosan modification by thiourea and rubeanic acid suggested in [97] enabled one to obtain **39S** and **44S** (Figure 3) derivatives with the degrees of substitution equal to 0.31 and 0.43, respectively, characterized with much higher sorption capacity toward Pt(IV) and Pd(II) ions (Table 4), which is provided by weak crosslinking and larger sorbent swelling ability due to low modification degree. The increase of the degree of substitution of thiocarbamoyl derivatives (**39S**, Figure 3) synthesized without using a linker up to 0.9 enabled one to produce materials with the highest sorption capacities toward Au(III) and Pd(II) ions [96]. Thiocarbonyl and dithiooxamide derivatives of chitosan were characterized by high sorption capacity, even at low degrees of substitution [30,31,87,96,97,100,127] (Table 4).

Mercapto derivatives are characterized with similar sorption properties toward Pd(II) ions and sorb more efficiently Pt(IV) ions [87] as compared to thiocarbonyl derivatives that results from higher donor properties of sulfur in the mercapto group than in the thiocarbonyl group (Table 4). Similar effect is observed for Ag(I) ions sorption [88,101].

At the same time, sorption properties are determined not only by the substituent nature and the functional groups content, but also by other material properties, in particular, degree of crystallinity and porosity [1,87], controlling the accessibility of sorption sires. For instance, microspheres of the chitosan mercapto derivative (**32S**, Figure 3) of a size of 6–30 µm with the sulfur content equal to 3.26 mmol/g (Table 4) had much lower sorption capacity (0.66 mmol Pt(IV)/g and 1.04 mmol Pd(II)/g [89]) as compared to derivatives with comparable degrees of substitution reported in [87]. 

Studies on *N*-(methylthiocarbamoyl) chitosan (**40S**, Figure 3) and N-(phenylthiocarbamoyl) chitosan (**41S**) showed that sorption of Cu(II) and Fe(III) ions on these derivatives depended on pH and is accompanied with the proton release, whereas sorption of Ni(II), Pb(II), Hg(II), and Cd(II) manifested virtually no dependence on pH, which indicates to formation of coordination bonds between metal and polymer [93]. Thus, the **40S** (Figure 3) derivative behaves as a monoatomic acid and a bidentate ligand. Differences in rows of preferential sorption of metal ions for **40S** and **41S** (Figure 3) derivatives enabled the authors to conclude on the effect of the structure of the substituent alkyl group on the derivatives selective properties.

Manifestation of selective properties toward Pb(II) ion, which is, in accordance with the Pearson principle, a softer acid as compared to Cu(II) and Cd(II) ions, was registered for dithiocarbamate chitosan derivatives (**46S**, Figure 3) [90] with the degree of substitution equal to 0.57. To increase the chitosan selectivity to Pb(II) ions, the authors of [129] suggested a combined approach consisting in preliminary templating O-carboxymethyl chitosan with Pb(II) ions followed by crosslinking using a polymer Schiff base thiourea/glutaraldehyde. As a result, the material with the sorption capacity toward Pb(II) ions equal to 2.02 mmol/g providing sufficiently high selectivity of Pb(II) removal in the presence of Cu(II), Zn(II), Cd(II), and Ni(II) ions was fabricated. A similar approach was applied to obtain materials selective to Ag(I) ions [128]. At the sorption capacity toward Ag(I) ions equal to 3.77 mmol/g, the fabricated material guaranteed the selective silver removal in the presence of Cu(II), Ni(II), Cd(II), Zn(II), and Ca(II) ions. Templating of chitosan derivatives (**32S,** Figure 3) by Ag(I) ions allowed a substantial increase of the sorption capacity toward Ag(I) ions and the selectivity of magnetic nanocomposites obtained on their basis in the presence of Cd(II), Zn(II), Pb(II), and Cu(II) ions [88].

In compliance with the Pearson principle, introduction of sulfur atoms into the chitosan structure substantially improves its sorption capacity toward Hg(II) ions having soft acid properties. The authors of [127] demonstrated that chitosan modification by thiocarbamoyl groups (39S, Figure 3) enabled one to extend significantly the pH range of efficient sorption of Hg(II) ions and to increase the chitosan capacity up to 2.3 mmol/g at pH 2. Here, the authors assume that sorption occurs through coordination of Hg(II) ions with sulfur atoms of thiocarbamoyl groups with possible participation of the amino group, since the available literature does not contain X-ray structural data on low-molecular complexes confirming participation of the thiourea amino group.

Mercapto derivatives of chitosan demonstrates high sorption capacities toward Hg(II) ions as well [62]. For example, the capacity of *N*-(2-hydroxy-3-mercapto)propyl chitosan (**32S**, Figure 3) with the sulfur content equal to 2.5 mmol/g attains value of about 3 mmol/g in the pH range 2.5–4.5, which indicates to the possibility of coordination of the Hg(II) ion by just one sulfur atom, not two as was assumed in [101]. Thus, in comparison with thiocarbonyl derivatives mercapto derivatives manifest better sorption capacity toward ions of both noble and transition metals, which is provided by higher donor properties of sulfur in the mercapto group than in the thiocarbonyl one. In case of transition metal ions, chelate effect has a significant influence on sorption properties. It is much more pronounced in case of **31S** and **32S** compared to **35S** and **39S** derivatives (Figure 3), so that the sorption capacities of **31S** and **32S** derivatives for Cu(II) and Hg(II) ions are higher (Table 4).

The presence of the strong acidic sulfo group (**38S** derivative) ensures the possibility of Hg(II) ions uptake from acidic solutions [98]. Formation of the chelating 2-sulfoethyl group (**37S**, Figure 3) is responsible for emergence of selective sorption of Ag(I) ions over Cu(II) ions [130]. The selectivity coefficient K_Ag/Cu_ at рН = 6.5 was equal to 20.

Least successful were attempts of bind hard acids (Eu(III) ions) using S-containing chitosan derivatives. Ambiguous data are also available for the efficiency of removal of Co(II) and Ni(II) ions related to acids with the intermediate hardness, according to the Pearson classification (Table 4). For the chitosan benzoyl thiourea derivative (**42S**, Figure 3) with the sulfur content equal to 2.2 mmol/g, the values of sorption capacity toward Co(II) and Eu(III) ions are equal to 29.47 and 34.54 mg/g, respectively [94]. Low sorption capacity values toward Co(II) and Ni(II) ions were also found for another chitosan derivative (**43S**, Figure 3) with a sulfur content of 2.8 mmol/g [95]. 

#### 2.2.4. P-Containing Chitosan Derivatives

Introduction of strong acidic functions (**48Р**, Figure 4) results in a significant increase of the ion-exchange capacity, but without gaining selectivity [103]. As seen from Table 5, both alkali-earth and transition metal ions are sorbed efficiently. 100% removal of Co(II), Cu(II), Ni(II), and Hg(II) ions is attained in the pH range from 2 to 6 [104]. The chelating derivative **49P** (Figure 4) has lower sorption capacity and selectivity toward transition metal ions [105].

## 3. Chitosan Derivatives with Linkers

### 3.1. Main Synthesis Methods 

#### 3.1.1. О-Containing Chitosan Derivatives

**Figure 5:**
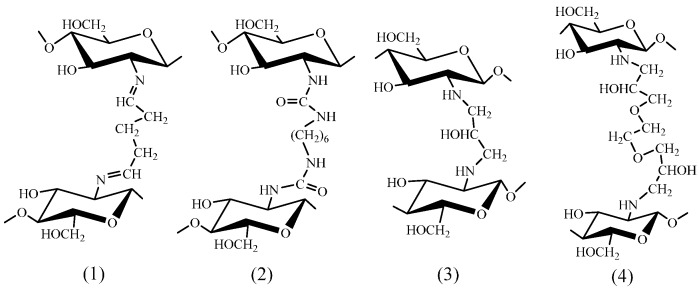
Structural fragments of crosslinked derivatives of chitosan obtained with glutaraldehyde (1), hexamethylene diisocyanate (2), epichlorohydrin (3), and ethylene glycol diglycidyl ether (4).

The simplest derivatives with linkers are crosslinked chitosans. In this case, chitosan functionalization proceeds via substitution and addition reactions (Figure 5).

**Figure 6:**
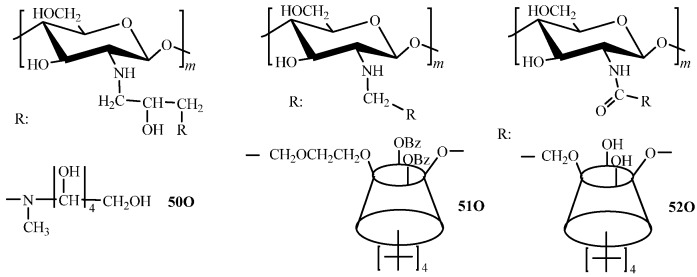
Structure of O-containing derivatives of chitosan with linker: **50O**—chitosan resin derivatized with *N*-methyl-d-glucamine; **51O**—calix[4]arene-crosslinked chitosan (5,11,17,23-tetra-*tert*-butyl-26,28-dibenzoyloxycalix[4]arene chitosan); **52O**—calix[4]arene-crosslinked chitosan (5,11,17,23-tetra-*tert*-butyl-26,28-dihydroxycalix[4]arene chitosan).

Functional derivatives with linkers are represented only by weak OH-acids (Figure 6) obtained via substitution (**50O**, **51O**, Figure 6) [56,57,58] and acylation reactions (**52O**, Figure 6) [131]. When the substitution reaction is used, derivatives with low degree of substitution, which does not exceed 0.3, are obtained. In case of acylation using the carbodiimide coupling reagent, a high degree of functionalization can be attained [131].

Crosslinking of hydroxylated chitosan derivatives can be performed using ethylene glycol diglycidyl ether [57]. Alternatively, functionalization reagent itself can act as a crosslinking agent [58,131].

#### 3.1.2. N-Containing Chitosan Derivatives

**Figure 7:**
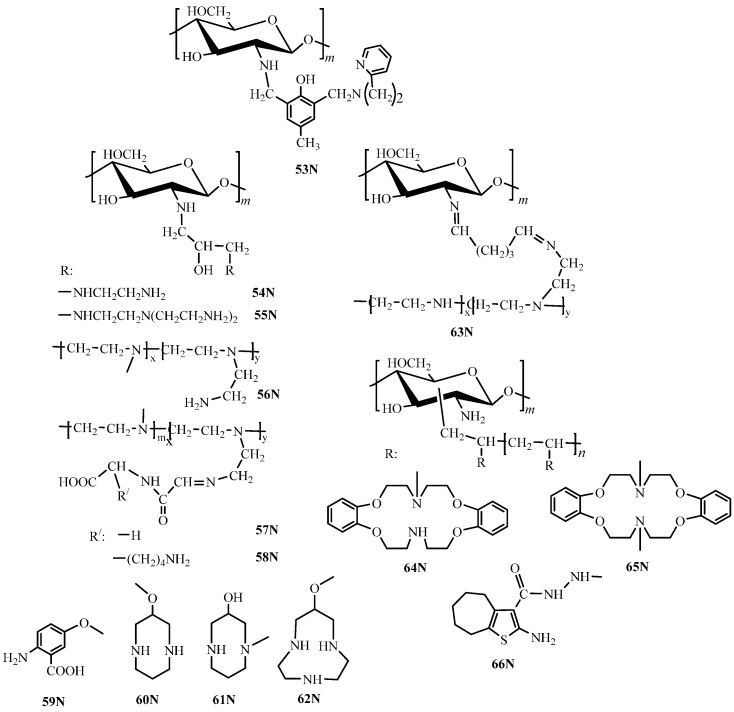
Structure of N-containing derivatives of chitosan with linker: **53N**—chitosan functionalized with 2[-bis-(pyridylmethyl)aminomethyl]-4-methyl-6-formylphenol; **54N**–ethylenediamine modified magnetic crosslinking chitosan; **55N**—chitosan-based resins modified with tris(2-aminoethyl)amine moiety; **56N**—polyaminated chitosan chelating resin; **57N**—glycine modified crosslinked chitosan resin; **58N**—l-lysine modified crosslinked chitosan; **59N**—chitosan resin functionalized with 2-amino-5-hydroxybenzoic acid; **60N**—1,5-diazacyclooctane chitosan; **61N**—3-hydroxy-1,5-diazacyclooctane chitosan; **62N**—3-hydroxy-1,5,8-triazacyclodecyl chitosan; **63N**—polyethyleneimine-grafted chitosan; **64N**—*N*-allyldibenzo-18-crown-6 crown ether crosslinked chitosan; **65N**—*N*,*N′*-diallyldibenzo-18-crown-6 crown ether crosslinked chitosan; **66N**—chitosan-grafted-poly(2-amino-4,5-pentamethylenethiophene-3-carboxylic acid *N*-acryloyl-hydrazide) chelating resin.

To obtain amino-alkylated derivatives, glutaraldehyde- or epichlorohydrin-based linkers are used in both addition and substitution reactions. This method is applied for chitosan functionalization with polyethyleneimine (PEI) using glutaraldehyde (**63N**, Figure 7) [87] or epichlorohydrin (**56N**, Figure 7) [113], which are simultaneously crosslinking and linker-forming agents, ethylenediamine (**54N**, Figure 7) [114,116]; tris(2-aminoethyl)amine (**55N**, Figure 7) [132], and 2-amino-5-hydroxybenzoic acid (**59N**, Figure 7) [67] through the hydroxypropyl linker. In some cases, chitosan preliminary crosslinked using ethylene glycol diglycidyl ether [113,116,132] or epichlorohydrin [61] is further functionalized. Functionalization of chitosan conjugate with PEI by aliphatic amino acids is performed using acylation reactions through formation of terminal carboxyl groups (**57N**, **58N**, Figure 7) [122,123].

There is a well-known example of the dipyridylmethyl derivative of chitosan (**53N**, Figure 7) functionalized through the dimethylphenolic linker using the reductive alkylation with subsequent crosslinking by glutaraldehyde [133].

Azacrown ethers of different structures (**60N**–**62N**, Figure 7) are used as non-aromatic heterocyclic fragments introduced into the chitosan using a linker [118,119,120,121,134,135,136]. For this purpose, the glycidyl ether of chitosan [120,135] or azacrown ethers [118,121,134] are obtained and then added to azacrown ethers or chitosan, respectively. The alternative method consists in block grafting of *N*-allyl derivatives of azacrown ethers to chitosan (**64N**, **65N**, Figure 7) [119,136]. In all cases, epichlorohydrin served as a crosslinking agent. The block polymerization with subsequent acylation were also used to obtain amino-thiophene derivative (**66N**, Figure 7) with the grafting percentage 173% [117].

#### 3.1.3. S-Containing Chitosan Derivatives

**Figure 8:**
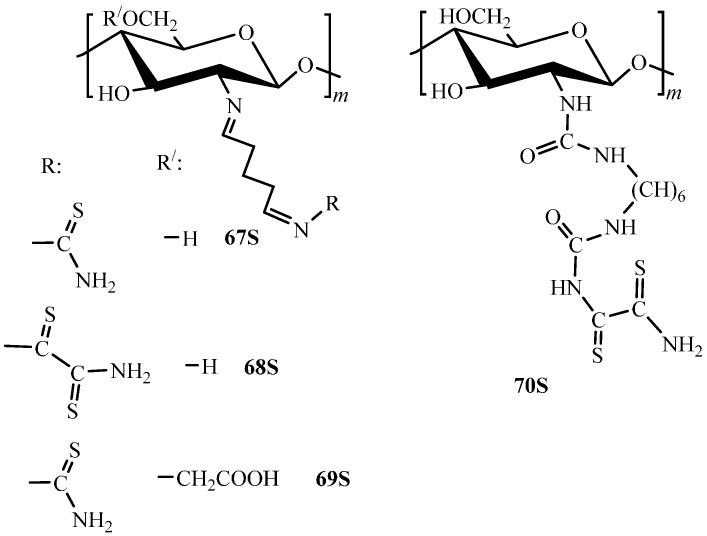
Structures of S-containing derivatives of chitosan with linker: **67S**—thiourea derivative of chitosan; **68S**—rubeanic acid derivative of chitosan; **69S**—thiourea-modified chitosan resin (thiourea-modified carboxymethyl chitosan); **70S**—sulfur derivative of chitosan (rubeanic acid derivative of chitosan cross-linking with hexamethylene diisocyanate).

To obtain thiocarbonyl derivatives, the pendant functionalization with thiourea [30,100,101,128,129] and dithiooxamide [30,31], and glutaraldehyde as both crosslinking and linker-forming agent is used. As a result, the following derivatives were obtained: thiourea derivatives of chitosan (**67S**, **69S**, Figure 8) and rubeanic acid derivative of chitosan (**68S**, **70S**, Figure 8) with the degree of substitution up to 0.3 and 0.2, respectively.

### 3.2. Sorption Properties

#### 3.2.1. О-Containing Chitosan Derivatives

Hydroxyl derivatives of chitosan (**14O**, **15O**, Figure 1; **50O**–**52О**, Figure 6) are characterized by compatible in the row sorption capacity (for example, toward Cu(II) ions [57]), but, in most cases, lower value in comparison with carboxyalkyl chitosans, that is expected because of less expressed donor properties of the hydroxyl group [58,59,131]—see Table 2. Here, conjugation of already formed calix[4]arene fragments to chitosan yields high selectivity of derivatives providing the efficient removal of Hg(II) [131] and Ag(I) [58] ions. Due to high affinity of a number of nonmetals to polyol compounds, hydroxyalkyl chitosan derivatives (**50О**, Figure 6) are highly efficient sorbents for B(III) removal [57].

#### 3.2.2. N-Containing Chitosan Derivatives

Studies of Hg(II) ions sorption by high-porosity crosslinked chitosan modified by polyethyleneimine (PEI) fragments of a molecular weight from 300 to 10,000 Da and the ratio between primary, secondary, and tertiary amino groups equal to 1:2:1 demonstrated [113] that modification resulted in the increase of both sorption capacity and sorption constant, thus corroborating higher affinity of the 56N derivative (Figure 7) to Hg(II) ions. Here, derivatives with the highest nitrogen content obtained through grafting PEI fragments of a molecular weight of 10,000 Da show the highest capacity in the рН range 2–8. Studies of sorption of 27 ions on 56N (Figure 7) derivative revealed the following selectivity row: Hg(II) > UO_2_(II) > Cd(II) > Zn(II) > Cu(II) > Ni(II), which is in rather good agreement with the stability of ammonia complexes of these metals in solution [113].

**Figure 9:**
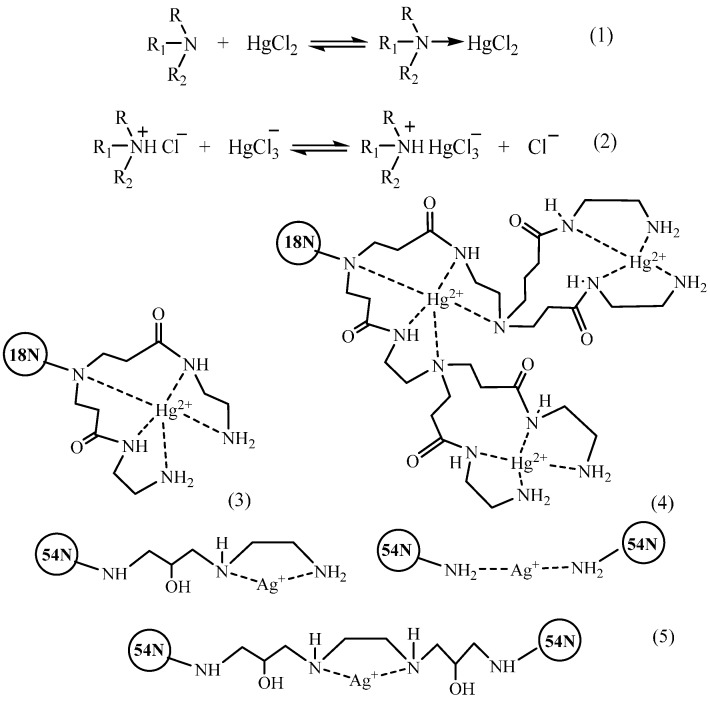
Suggested structures of aminated chitosan derivatives complexes with transition metal ions—1, 2 [113]; 3, 4 [70]; 5 [116].

The suggested sorption mechanism depends on the nature of the sorbed metal as well as on the pH value determining the degree of protonation of polymer amino groups [113,116]. For example, in chloride solutions in the neutral pH range, the main form of Hg(II) is the neutral HgCl_2_ complex, which, according to the authors’ opinion, most probably interacts with the PEI-chitosan conjugate in accordance with the reaction 1 (Figure 9). Sufficiently high values of the sorption capacity toward mercury in the acidic medium result from the possibility of the anion-exchange reaction 2 (Figure 9). The possibility of formation of chelate compounds of amino derivatives of chitosan with Hg(II) ions through involvement of four or five nitrogen atoms in metal ion coordination (structures **3** and **4**, Figure 9) was assumed for chitosan functionalized by amino-terminated hyperbranched polyamidoamine polymers (**18N**, Figure 2) [70] and diethylenetriamine derivatives (**55N**, Figure 7) [132]. In this case, the sorption capacity did not increase along with the increase of the dendrimer branching degree and, thus, the number of nitrogen atoms (as could be expected), but decreased due to steric hindrances and higher crosslinking degrees for derivatives with higher degrees of oligomerization.

In spite of the similarity of electron-donor properties of functional groups of the native chitosan and its amino derivatives, steric effects and differences in substituents structures could affect substantially the selectivity of metal ions removal. For instance, as was established in [116], the affinity of ethylenediamine derivatives of chitosan (**54N**, Figure 7) appeared to be higher to Ag(I) ions than to Cu(II) ions in the рН range 1–8, which was related to the possibility of formation of a five-membered chelate ring (structure 5, Figure 9), whereas the sorption capacity of the unmodified chitosan toward Ag(I) was noticeably lower than that toward Cu(II) ions—see Table 1. Depending on рН, it was also possible to use the **54N** derivative in quantitative removal of Sn(II) ions at pH 4–9, Bi(III) ions at pH 3–9, and Th(IV) ions pH 6–9 [116]. Reduction of the chelating ability and extra spatial hindrances of the amino group (**21N** derivative, Figure 2) result in a substantial decrease of the sorption capacity [68].

One of the highest sorption capacities toward Cu(II), Co(II), and Ni(II) ions reported in the literature for the chitosan derivatives are related to chitosan-grafted-poly(2-amino-4,5-penta-methylenethiophene-3-carboxylic acid *N*-acryloyl-hydrazide) chelating resin (**66N**, Figure 7), whose structure allows assuming the possibility of formation of chelate compounds of different compositions with involvement of amino and hydrazide groups [117]. 

The decrease of the sorption capacity along with the increase of crosslinking degree and polymer framework hardness was demonstrated for imidazolyl chitosan derivatives [82]. High crosslinking degree could be also responsible for low sorption capacity of conjugates of PEI-chitosan with amino acid (**57N** and **58N**, Figure 7) [122,123]. 

Significant increase of the chitosan selectivity can be attained through covalent introduction of azacrown ethers [118,119,120,121,134,135,136], although the sorption capacity of this type of sorbents is rather low in comparison with other derivatives. The possible explanations of low sorption capacity are high crosslinking degree [118] and significant increase of hydrophobicity providing lower degree of sorbents swelling ability. Changes in the chemical structure of azacrown ethers have virtually no effect on the sorption capacity; however, despite low sorption capacities one attains high selectivity of sorption of Cu(II) [118] and Hg(II) ions [134] for the **60N** (Figure 7) derivative, Ag(I) ions [119,120,121,135,136] for **61N**, **62N**, **64N**, and **65N** derivatives, and Pd(II) ions [119,136] for **64N** and **65N** derivatives (Figure 7). Unfortunately, lack or total absence of quantitative parameters, which are required to systematically characterize the composition and structure of a number of synthesized sorbents [68,114,122,123,132], complicates the analysis of synthesis method-structure-sorption properties correlations for new materials.

#### 3.2.3. S-Containing Chitosan Derivatives

Introduction of thiourea and dithiooxamide using glutaraldehyde as a linker (**67S** and **68S**, Figure 8) derivatives, respectively, allowed increasing the chitosan sorption capacity up to 3.24 mmol Pd(II)/g [30] and 1.77 mmol Pt(IV)/g [100].

Rubeanic acid derivatives of chitosan are characterized by larger sorption capacities even at low degrees of substitution [30,97] (Table 4). As was shown for sorption of Pd(II) ions [30], the sorption constant and, therefore, the affinity change in the row of materials: chitosan< **67S** < **68S**. Comparison of the sorption data for chitosan derivatives containing the thiourea residue [30,96,97,137] demonstrates that, independently of the synthesis method, the derivatives sorption capacity toward Pt(IV) ions is in average 1.5-2-fold lower than that toward Pd(II) ions, which is related to structural peculiarities and different stability in solution of chloro complexes of these metals [137]. Studies of the Pt(IV) and Pd(II) ions sorption from mixed solutions on chitosan crosslinked by glutaraldehyde and the **67S** derivative [87] showed that these ions were bound to the same sorption sites. In spite of the fact that the affinity of both native and modified chitosan is significantly higher toward Pd(II) ions, the selectivity appeared to be insufficient to separate Pt(IV) and Pd(II) under both static and dynamic conditions.

At the same time, comparative analysis of sorption properties of chitosans modified with [30,31,87,100] or without [96,97,127] linker demonstrates that the pendant modification by thiocarbonyl compounds providing higher mobility of the grafted functional fragment relatively to the polymer chain enables one to fabricate materials with high sorption capacity at low degrees of substitution. Synthesis of high-substituted derivatives using linkers must be impossible, since at large excess glutaraldehyde will crosslink macromolecules stronger, thus increasing the crosslinking degree and decreasing the number of amino groups accessible for functionalization.

## 4. Mechanisms of Interaction of Chitosan and its Derivatives with Metal Ions

### 4.1. Interaction of Chitosan and Its Derivatives with Metal Cations

**Figure 10:**
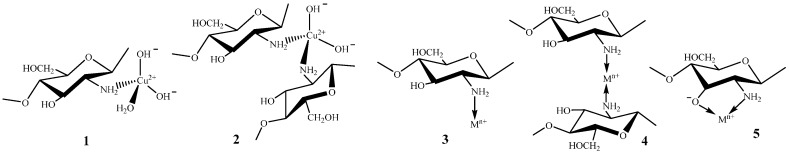
Suggested structures of complexes with chitosan: **1**, **2**—adapted from [142]; **3,** **4**, **5**—adapted from [148].

The mechanism of chitosan interaction with transition metal cations was discussed in sufficient detail in experimental [138,139,140,141,142,143] and theoretical [144,145,146,147] works as well as in a number of reviews [1,2,15]. As a rule, two possible models of binding metal ions by chitosan are considered: the “bridge” model describing the metal ion coordination with several amino groups belonging to different glucosamine units of the same or different polymeric chains (structures **2** and **4**, Figure 10) [138] and the “pendant” model, in which the metal ion is coordinated with just one amino group (structures **1** and **3**, Figure 10) [141]. It is evident that the most active coordination center for transition metal ions is the nitrogen atom of the chitosan amino group, while many authors investigated the possibility of participation of other chitosan functional fragments in complex formation using quantum chemistry methods—acetamide [145,146] and hydroxyl [145,146,147] groups as well as the oxygen atom of the glycoside bond [147].

A majority of the researchers agree that the acetamide group does not participate in complex formation that is supported by quantum chemistry calculations [146] and experimental data [138,143]. The authors of [147] concluded that Cu(II) and Ni(II) were coordinated by one or two amino groups of the disaccharide chitosan fragment, the oxygen atom of the glycoside bond, and the oxygen atom of the hydroxyl groups in positions C-6 or C-3. In opposite, the authors of [146] showed that the chitosan monomer (glycosamine) could not serve as a bidentate ligand, and the coordination sphere of the Cu(II) ion bonded to the amino group was completed with water molecules and OH^−^ ions. At the same time, some experimental data [139,140,141] corroborate participation of chitosan hydroxyl groups in coordination of transition metal ions.

No less debatable is the problem of the model of complex formation. The “pendant” model suggested in experimental works [140,141] found corroborative data in the calculation work [146], which, on the other hand, did not exclude the possibility of Cu(II) coordination ions in compliance with the “bridge” model. The authors of [28,144], devoted to binding of Hg(II) and Pb(II) ions to chitosan, considered only the “bridge” model of complex formation. The latter model was also recognized as the most probable using the results of density functional theory calculations [145]. The possibility of realization of both models of complex formation with Cu(II) ions was suggested in [142] through demonstration that, depending on pH, “pendant” (structure **1**, Figure 10) or ”bridge” (structure **2**, Figure 10) complex can be the most stable.

As was shown using the method of potentiometric titration for calculation of constants of complexes stability, all three types of structures (**3**, **4**, and **5**, Figure 10) were possible for binding of native chitosan with Co(II) [129] and Ni(II) [148] ions. Depending on the metal/ligand molar ratio, coordination of the Co(II) ion can be realized according to “bridge” (structure **4**, Figure 10) or “pendant” (structure **3**, Figure 10) models as well as through formation of a five-membered ring with participation of the hydroxyl group in position С-3 (structure **5**, Figure 10). In opposite, for Zn(II) [149] and Mn(II) [148] ions, coordination is possible only with participation of amino groups (structures **3** and **4**, Figure 10).

To sum up, one can conclude that the probability of realization of any model of transition metal ions coordination depends on metal ion nature, metal/ligand ratio, and pH. Extra possibilities of chelating metal ions emerge along with introduction of new functional fragments into the chitosan structure.

Ni(II) ions complexation with chitosan and its *N*-heterocyclic derivatives—*N*-(2-pyridylethyl) chitosan (**2-PEC**), *N*-(4-pyridylethyl)chitosan (**4-PEC**), and *N*-(5-methyl-4-imidazolyl)methyl chitosan (**IMC**) was investigated in [150]. It was shown that in most cases formation of “pendant” complexes was more favorable. Nitrogen atoms of chitosan and its *N*-heterocyclic derivatives played a governing role in Ni(II) binding and the degree of charge transfer from the ligand to the central ion in the complexes correlated with the complex stability. A row of complexes stability depending on the type of functional substitute in chitosan macromolecules (**IMC** ≈ **2-PEC** > chitosan > **4-PEC**) was in a good agreement with experimental data obtained from the Ni(II) ion sorption isotherms on chitosan, **2-PEC**, **4-PEC** and **IMC**.

The sorption capacity of both chitosan and its derivatives does not reflect to full extent the true polymer affinity to ions of specific metals, since it depends significantly on material form, synthesis conditions, and crosslinking degree. It is evident that the most informative are the data on complexes stability and formation energies determined by calorimetry methods or calculated by quantum chemistry methods. Unfortunately, the data of these types are very limited in the literature, so that in most cases one can perform just qualitative or semi-quantitative comparison of the complexing ability of the native chitosan and its derivatives differing by functional fragments.

**Figure 11:**
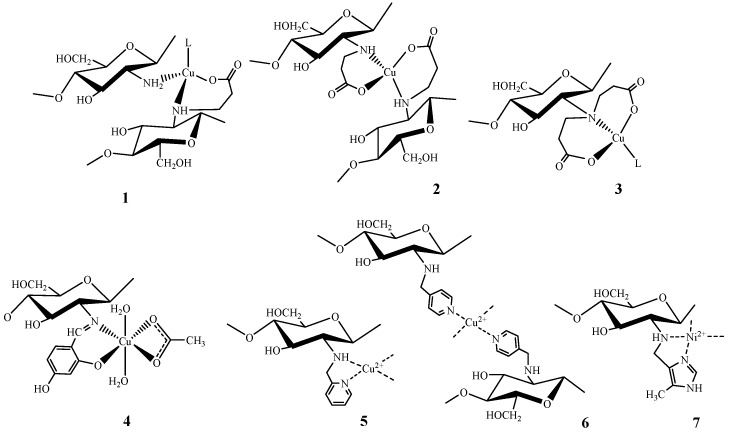
Suggested structures of complexes with *N*-(2-carboxy)ethyl chitosans, **1**–**3**—from [46]; with *N*-(4-hydroxysalicylidene) chitosan, **4**—from [151]; with *N*-(2-pyridyl)methyl and *N*-(4-pyridyl)methyl chitosan), **5,** **6**—from [115]; with *N-*(5-methyl-4-imidazolyl)methyl chitosan, **7** from [82].

Carboxyalkyl chitosans appear to be the only exception, for which the literature data allow tracing changes in the complexes stability in dependence on the degree of substitution [46] and the metal ion nature [14]. The increase of the chitosan amino group basicity as a result of carboxyalkylation [14,46] and the possibility of formation of five- and six-membered chelate rings in the case of carboxymethyl and carboxyethyl chitosan, respectively (Figure 11), results in a substantial increase of the constants of stability of complexes with transition metals, as compared to the native chitosan (Table 6).

As seen on the example of *N*,*O*-carboxymethyl chitosan, the stability constants change in accordance with the Irving-Williams row (Mn(II) < Co(II) < Ni(II) < Cu(II) > Zn(II)) taking into account the increased stability of the complex with Cu(II) due to the Jahn-Teller effect. The established dependence of the logarithm of stability constant on the metal electronegativity corroborated the increase of the covalent character of the metal-ligand bond and, therefore, the chelate stability, along with the increase of the metal electronegativity [14].

Analysis of the dependence of the stability constant for *N*-(2-carboxy)ethyl chitosan complexes with Cu(II) on the degree of modification [46] showed that derivatives with degrees of substitution of 0.42 and 0.92 formed complexes of the bridge type CuL_2_ (structures **1** and **2**, Figure 11), whereas high-substituted derivatives (degree of substitution 1.61) with high contents of fragments of iminopropionic acid coordinate Cu(II) ions predominantly with formation of less stable pendant complex (structure **3**, Figure 11). It is assumed here that the hydroxyl group in С-3 position does not participate in coordination due to steric limitations.

It is worth mentioning that the possibility of formation of a chelate ring ensures higher stability of the Cu(II) complex with carboxyethyl chitosan, as compared to that with methyl-2-amino-2-deoxy-D-glucopyranoside, which is a ligand with higher basicity. Here, the stability constants of Cu(II) complexes with chelate-forming low-molecular ligand β-alanine are more than two orders of magnitude higher than for complexes with a ligand with lower—carboxyethyl chitosan [46]. Significantly lower basicity of *N*-substituted imines (as compared to amines) is a probable explanation of low constants of stability of complexes of Co(II), Cu(II), Ni(II), and Zn(II) with *N*-(4-hydroxysalicylidene) chitosan (structure **4**, Figure 11) [151].

The possibility of formation of stable five- and six-membered chelate rings with participation of nitrogen atoms from the main chitosan chain and substituent is realized for *N*-heterocyclic chitosan derivatives. Unfortunately, the available literature does not contain data on the stability of such complexes. Some ideas on the effect of chelating on the complex stability can be derived from the results of studying the interaction of Cu(II) ions with *N*-(2-pyridyl)methyl and *N*-(4-pyridyl)methyl chitosan: the former is a bidentate chelate-forming ligand (structures **5** and **6**, Figure 11) [115]. The constant of sorption on *N*-(2-pyridyl)methyl chitosan is 2.5-fold higher in comparison with that on the pyridyl derivative not forming chelates. In case of imidazole-containing derivatives, high sorption capacity toward Ni(II) ions can be also attributed to possibility of formation of a five-membered chelate ring (structure **7**, Figure 11) [82].

**Figure 12:**
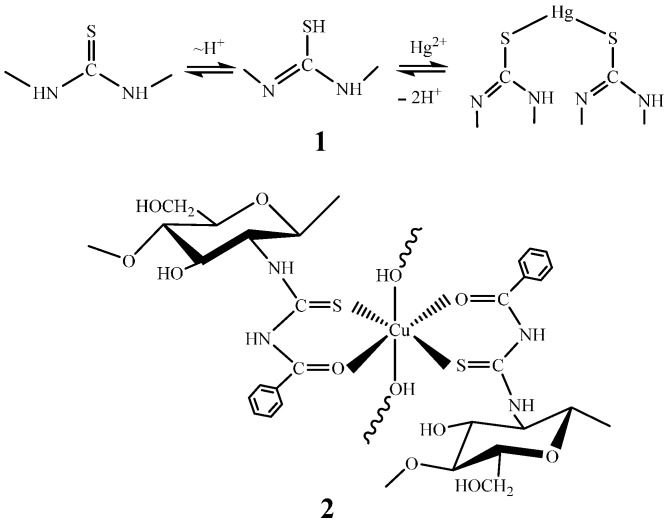
Suggested structures of complexes with thiocarbamoyl derivatives of chitosan, **1**—adapted from [126]; with chitosan benzoyl thiourea derivative, **2**—adapted from [94].

In case of mercapto chitosan derivatives, possible structures of the formed complexes upon sorption of transition metal ions cannot be found in the literature. Nevertheless, one can assume the formation of the sulfide bond, since the affinity to Cu(II) increases significantly in comparison with the initial chitosan or its amino derivative [84], in accordance with the Pearson principle. Thiocarbonyl chitosan derivatives are often considered as monodentate ligands [30,96,97,126] which are also capable to form the sulfide coordination bond (structure **1**, Figure 12) that complicates desorption in case of Hg(II) ions [126]. For the chitosan benzoyl thiourea derivative, the authors suggest the formation of chelate rings (structure **2**, Figure 12) [94].

### 4.2. Interaction of Chitosan and its Derivatives with Metal Anions

Although a majority of studies on sorption properties of chitosan and its derivatives are devoted to interaction of polymeric ligands with metal cations, sorption of anionic metal forms is of no less practical interest. Such studies are the most extensive for chloro complexes of noble metals, which is related to both—recovery of valuable components from technological solutions [31] and development of techniques for analytical pre-concentration of noble metals [152]. 

**Figure 13:**
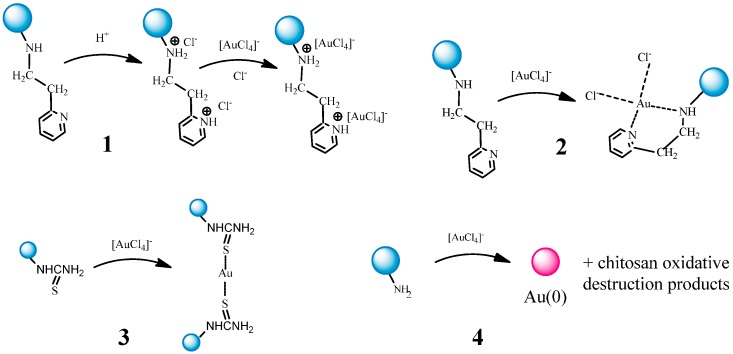
Possible mechanisms of metal anion complexes interactions with chitosan and its derivatives: 1—ion-exchange; 2 and 3—coordination; 4—reduction.

Depending on the nature of metal ion and structure of functional substituent in chitosan derivatives sorption of metal anion complexes can proceed via one of the following mechanisms—ion-exchange (structure **1**, Figure 13), metal ion coordination (structures **2** and **3**, Figure 13), and reduction (structure **4**, Figure 13).

Since one of the possible (sorption) mechanisms is based on electrostatic interaction between anionic complex and oppositely charged chitosan amino groups, the solution pH value determining the degree of molecule protonation is one of the key factors affecting the value of sorption capacity. Taking into account that pK_a_ of chitosan varies from 6.45 to 6.90 with changes in the degree of deacetylation from 94.8 to 29.4% [153], one can conclude that an overwhelming majority of chitosans under study are fully protonated at рН < 4.5. However, at very low pH values, competition between background anions and anionic metal complexes for sorption sites results in a dramatic decrease of the sorption capacity [29].

The factors affecting the sorption of molybdate- and vanadate-ions as well as anionic copper complexes from citrate solutions were considered in sufficient detail in [1,154,155]. For example, it was demonstrated that the increase of the sorption capacity of chitosan toward molybdate- [1] and vanadate-ions [155] at pH 3 was related to formation of polynuclear multiply charged complexes. Changes in the form of metal presence from cationic to anionic form could also result in changes in the sorption mechanism from chelating to ion exchange mechanism [154]. Thus, the optimal pH value for anion sorption is determined by chitosan amino groups’ protonation degree and charge of metal anion.

Chemical modification of chitosan to increase the efficiency of removal of anionic forms of metals consists in ensuring the material insolubility in acidic media provided via crosslinking [29] and the increase of sorption capacity and selectivity via introduction of functional fragments increasing the number of electron-donor nitrogen atoms in the macromolecule [87] or high-affinity functional fragments, for example, sulfur-containing groups for removal of noble metal ions [31,96,97,100].

**Figure 14:**
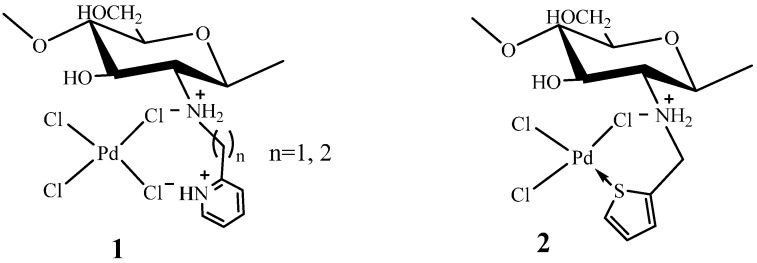
Suggested structures of quasi-chelates with Pd(II) anions.

Introduction of extra electron-donor nitrogen atoms into the chitosan structure, for example, through grafting the fragments of polyethyleneimine [156], ethylenediamine [157], imidazole [82], and pyridine [75,78] can significantly change chitosan sorption capacity and selectivity toward metal anions. The differences in pK of primary, secondary, and tertiary amino groups in grafted polyethyleneimine fragments result in broadening of the pH-range of the efficient sorption [156]. Noticeable changes in the metals affinity to chitosan and its derivatives were mentioned in [78]. For instance, the constants of sorption of Pt(IV) and Pd(II) ions increase along with the increase of the derivative degree of substitution, whereas the decrease of sorption constants along with the increase of the HCl concentration in the range 0.1–1 M for *N*-2-(2-pyridyl)ethyl chitosan was less significant, as compared to the unmodified chitosan. It is possible that the increase of the stability of complexes with polyvalent Pt(IV) and Pd(II) ions is related to quasi-chelating of the ionic pair (structure **1**, Figure 14). The possibility of realization of the mixed sorption mechanism (ion exchange and chelate formation) (structure **2**, Figure 14) with participation of sulfur atoms from the substituent was not excluded in [75,157].

Analysis of sorption properties of N-containing chitosan derivatives—*N*-2-(2-pyridyl)ethylchitosan (**2-PEC**), *N*-2-(4-pyridyl)ethylchitosan (**4-PEC**), and *N*-(5-methyl-4-imidazolyl)methylchitosan (**IMC**) [78,82,158]—revealed the following interesting trends [158]: (i) increase of affinity toward Pt(IV) and Pd(II) ions from 0.1M HCl solutions for all derivatives in comparison with chitosan and changes of their sorption capacities the row **2-PEC** > **4-PEC** > **IMC** > chitosan; (ii) unexpectedly high sorption capacity of chitosan for Au(III) ions (**2-PEC** > chitosan > **IMC** ≥ **4-PEC**) that distorts the row of derivatives affinity found for Pt(IV) and Pd(II) ions. 

The second trend shows that increased number of Lewis base sites due to introduction of electron donor N-atoms of functional fragments does not necessarily leads to higher sorption capacity toward Au(III) ions. Thus another mechanism different from ion-exchange can contribute to Au(III) sorption on chitosan and its N-derivatives. Recent findings on the mechanism of Au(III) reduction by chitosan [159] showed that coordination of Au(III) ions by chitosan promoted hydrolysis of glyosidic bonds and subsequent reduction of Au(III) to Au(0) by hydrolysis products. Most likely, this mechanism is valid for all N-derivatives of chitosan, although the strength of complexes formed by Au(III) and pyridylethyl or imidazolylmethyl moieties can differ significantly from that of Au(III)-chitosan complex that leads to lower hydrolysis rate and, thus, to less efficient reduction of Au(III) ions. It was found that sorption of Au(III) and Pt(IV) by chitosan, **2-PEC**, **4-PEC**, and **IMC** was accompanied by complete or partial metal reduction. The ratio between forms in different oxidation state in sorbent phase depends on the type of derivative with the highest content of reduced species—Au(0) and Pt(II) found in chitosan [158]. Taking into account literature data on Au(III) reduction to Au(0) in the process of sorption on polystyrene-supported glucosamine resin [160], chitosan [159] and other *N*-containing derivatives of biopolymers [161,162], one can suggest that formation of elemental gold was the most probable reason for high sorption capacity of unmodified chitosan for Au(III) ions.

Since the sorption mechanism (ion-exchange, chelation, or reduction) significantly affects the efficiency of metal ion elution after the sorption, the difference in elution efficiency of Pd(II) and Au(III) ions adsorbed via ion-exchange and reduction mechanism, respectively, can be used for quantitative separation of these ions from multicomponent solutions [163].

In accordance with the Pearson principle, introduction of sulfur-containing fragments into the chitosan structure imparts it with weak base properties that increases substantially its affinity to noble metal ions and ensures the possibility of their coordination with participation of sulfur and nitrogen atoms. Changes in the mechanism of noble metal ions sorption on sulfur-containing derivatives is illustrated the most clearly by changes in the character of the dependence of sorption properties on pH and the presence of competing ions.

The optimal pH value for the recovery of noble metal ions by chitosan and sulfur-containing derivatives is about 2–3 and depends on the type of functional substituent [31,93,100]. Along with the pH increase, the sorption capacity decreases dramatically for the crosslinked unmodified chitosan and just insignificantly for sulfur-containing derivatives [31]. Studies of the effect of competing anions (sulfates, nitrates, and chlorides) on the sorption properties of thiocarbamoyl chitosans with the degree of substitution equal to 0.7–0.83 showed that, unlike the native chitosan, the sorption capacity of derivatives toward Pt(IV) and Pd(II) ions decreases just insignificantly in the presence of these anions [127]. Here, it is worth mentioning that the effect of anions on materials sorption properties is to a great extent determined by their nature. For instance, introduction of chloride in some specific concentration range promoted the increase of noble metals sorption due to formation of anionic chloro complexes that efficiently participate in the reaction of ion exchange with chitosan [30]. In opposite, introduction of sulfate ions results in a significant decrease of the sorption capacity of the native chitosan that is related to the specific interactions between sulfate ions and chitosan resulting in changes of the polymer crystallinity rather than to the increase of solution ionic strength [1,30]. Although the possibility of formation of coordination compounds with noble metals is not excluded for the native chitosan [30], in most cases anion exchange considered as the main driving force for metal anions sorption, whereas weak dependence of the sorption properties of sulfur-containing derivatives on pH and competing ions concentrations indicates to predominance of the coordination mechanism of interaction.

The issue about the structure of noble metals complexes with sulfur-containing chitosan derivatives remains unresolved. The authors of [30] suggested on the basis of literature data on noble metals complexes formation with nitrogen and sulfur-containing polymers that the most probable model of Pd(II) ions binding by chitosan with grafted fragments of thiourea and dithiooxamide corresponds to the stoichiometric ratio 2:1 (for N-Pd and S-Pd). However, the literature data presented in this work and [96] demonstrate, in some cases, a superequivalent sorption of noble metals, which indicates to more complex composition and limited applicability of this model.

Chelating with subsequent reduction is also a possible mechanism of sorption on S-containing chitosan derivatives. Reduction of platinum ions until the most stable forms at lowest oxidation degrees was observed during sorption on many S- and N,S-containing heterochain synthetic polymers [164]. For sulfur-containing chitosan derivatives, reduction of Au(III) until Au(I)/Au(0) and of Pt(IV) until Pt(II) in the sorbent matrix was registered using the method of X-ray photoelectron spectroscopy [96].

## 5. Conclusions

Polyfunctional nature of chitosan determines one of the highest among natural polysaccharides metal binding ability, which enables its application as a polymer ligand not only for recovery, separation, and concentration of metal ions, but also as a matrix component of metal-containing catalysts, chromatographic substrates, optical devices, and antimicrobial and other medicinal drugs. At the same time, the native chitosan does not meet a wide range of requirements for polymers of polyfunctional applications, which limits the possibilities of its use in many promising fields.

Targeted chemical modification is the only efficient tool that allows fine adjustment of the chitosan Lewis basicity and chelating ability not only by introduction of functional groups that are different in donor properties from amino and hydroxyl groups, but also through formation of spatial structures of specific structure. Although one can predict selectivity rows of the derivatives using stability constants of the corresponding low molecular weight ligands, the synthetic procedures—modification with linker or without it, crosslinking conditions—play a significant role in mobility and steric accessibility of functional fragments and, as a result, in the sorbent selectivity.

In spite of certain progress in synthesis and demonstration of prospects of chitosan chemical modification, further development in this direction is impossible without deeper understanding of the “structure-properties” correlation and systematic studies of the effect of functional fragments structure and degree of substitution on the stability of complexes of chitosan derivatives with metals. This information allows predicting the chitosan behavior in biological and technological systems in the presence of other ligands, developing the methods of synthesis of high-selectivity derivatives, and optimizing properties of chelating flocculants and polymeric ligands for targeted synthesis of nanoparticles and hybrid materials.

## Figures and Tables

**Table 1:** Structure and sorption properties of native chitosan.

Crosslinking Reagent	MW, kDа	Degree of Acetylation	Sorption			Ref.
			Ion	pH	Capacity, mmol/g	
–	n.d.	0.47	Cu(II)	6	0.81	[18]
–	hight	0.15		4.2	2.67	[19]
–	n.d.	0.40		6	1.26	[20]
–	500	0.44		6	1.27	[17]
Glutaraldehyde	500	0.44		6	0.94	[17]
Epichlorohydrin	500	0.44		6	0.98	[17]
Ethylene glycol diglycidyl ether	500	0.44		6	0.72	[17]
Epichlorohydrin	hight	n.d.		3–5	1.259	[21]
Epichlorohydrin	n.d.	0.1		5	0.71	[22]
Epichlorohydrin	n.d.	0.25		6	0.56	[23]
Hexamethylene diisocyanate	n.d.	0.12		7	2.96	[24]
Epichlorohydrin	n.d.	0.1	Ni(II)	5	0.595	[22]
Glutaraldehyde	410	0.13		3.5–5.5	1.3	[25]
Formaldehyde	750	0.45	Ag(I)	7.6	0.31	[26]
Epichlorohydrin	hight	n.d.	Cr(VI)	5	1.5	[21]
Ethylene glycol diglycidyl ether	n.d.	0.15	Pb(II)	4	0.46	[27]
–	n.d.	0.14		7	0.18	[28]
–	n.d.	0.40		6	0,283	[20]
Epichlorohydrin	n.d.	0.25		7	0.16	[23]
–	n.d.	0.14	Hg(II)	8	0.0342	[28]
–	n.d.	0.40		4	0.546	[20]
Epichlorohydrin	n.d.	0.1	Cd(II)	5	0.332	[22]
Formaldehyde	750	0.45		7.4	3.74	[26]
Epichlorohydrin	n.d.	0.1	Co(II)	5	0.172	[22]
–	n.d.	0.40	Zn(II)	6	0.72	[20]
Glutaraldehyde	410	0.13		3.5–5.5	1.5	[25]
Epichlorohydrin	n.d.	0.25		7	0.15	[23]
Glutaraldehyde	125	0.13	Pd(II)	0.01 М HCl	1.53	[29]
Glutaraldehyde	125	0.13		2	2.44	[30]
Glutaraldehyde	125	0.13	Au(III)	1.6	2.9	[31]
Glutaraldehyde	125	0.13	Pt(IV)	2	1.58	[32]

**Table 2:** Structure and sorption properties of O-containing chitosan derivatives.

Chitosan Derivative	MW, kDа	Degree of Acetylation	Degree of Modification	Sorption			Ref.
				Ion	pH	Capacity, mmol/g	
**1O**	600	0.1	0.96	Cu(II)	5	2.56	[39]
**1O**	300	0.1	n.d.		5	2.05	[40]
**8O**	n.d.	0.1	0.42		5	2.13	[22]
**12O**	n.d.	0.12	n.d.		7	2.67	[24]
**13O**	n.d.	0.49	0.51		7	0.93	[18]
**7O**-Pb(II) templated	600	0.1	0.6		5.8	2.23	[53]
**6O**	318	0.26	157%		5.5	2.30	[51]
**50O**	n.d.	n.d.	0.25		6.5	1.7	[57]
**14O**	n.d.	0.25	0.22		7	0.9	[59]
**3O**	250	0.18	0.91		7	1.37	[47]
**1O**	30.6	0.16	0.91		5.5	2.71	[54]
**2O**	29.3	0.69	0.83		5.5	2.54	[54]
**14O**	n.d.	0.25	0.22	Ni(II)	7	0.29	[59]
**8O**	n.d.	n.d.	1.0		2	2.1	[107]
**9O**	n.d.	n.d.	0.22		2	2.0	[107]
**8O**	n.d.	0.1	0.42		5	1.34	[22]
**8O**	282.5	0.15	1.4 mmol/g		2.1	1.21	[49]
**9O**	282.5	0.15	0.96 mmol/g		2.1	0.90	[49]
**7O**-Pb(II) templated	600	0.1	0.6		5.8	2.08	[53]
**51O**	n.d.	0.2	0.23		5	0.6	[58]
**8O**	n.d.	0.1	0.42	Co(II)	5	1.28	[22]
**8O**	282.5	0.15	1.4 mmol/g		2.1	1.07	[49]
**9O**	282.5	0.15	0.96 mmol/g		2.1	0.83	[49]
**7O**-Pb(II) templated	600	0.1	0.6		5.8	2.20	[53]
**7O**-Pb(II) templated	600	0.1	0.6	Zn(II)	5.8	1.8	[53]
**1O**-Zn(II) templated	600	0.1	0.96		5.99	1.98	[52]
**4O**	490	0.05	n.d.		6	0.41	[48]
**5O**	490	0.05	n.d.		6	0.58	[48]
**14O**	n.d.	0.25	0.22		7	0.70	[59]
**1O**	545	0.1	1.78	Sr(II)	4	1.13	[42]
**8O**	n.d.	0.1	0.42	Pb(II)	5	1.28	[22]
**10O**	n.d.	0.15	n.d.		4	1.42	[27]
**7O**-Pb(II) templated	600	0.1	0.6		5.8	1.68	[53]
**8O**	n.d.	0.1	0.42	Cd(II)	5	1.29	[22]
**7O**-Pb(II) templated	600	0.1	0.6		5.8	2.1	[53]
**14O**	n.d.	0.25	0.22	Cd(II)	7	0.68	[59]
**7O**-Pb(II) templated	600	0.1	0.6	Hg(II)	5.8	1.75	[53]
**1O**	60	0.16	0.91	Pd(II)	4	0.0096	[44]
**2O**	250	0.75	0.81		4	0.0025	[44]
**51O**	n.d.	0.2	0.23		2	0.74	[58]
**1O**	600	0.16	0.91	Pt(IV)	4	0.0059	[44]
**2O**	250	0.75	0.81		4	0.013	[44]
**1O**	42	0.16	0.91	Au(I)	4	0.19	[108]
**2O**	250	0.75	0.81		4	0.06	[108]
**1O**	500	0.13	n.d.	Au(III)	6	0.17	[45]
**51O**	n.d.	0.2	0.23	Ag(I)	5.3	0.81	[58]
**11O**-Th(IV) templated	n.d.	n.d.	n.d.	Th(IV)	3	0.264 × 10^−3^	[50]
**50O**	n.d.	n.d.	0.25	B(III)	6.5	2.1	[57]
**15O**	n.d.	0	0.64		11	0.4	[56]
**15O**	n.d.	0	0.64	Ge(IV)	4	1.4	[56]
**15O**	n.d.	0	0.64	Te(IV)	10	0.4	[56]

**Table 3:** Structure and sorption properties of N-containing chitosan derivatives.

Chitosan Derivative	MW, kDа	Degree of Acetylation	Degree of Modification (N_tot_, mmol/g)	Sorption			Ref.
				Ion	pH	Capacity, mmol/g	
**26N**	n.d.	0.24	0.85	Cu(II)	7.6	1.63	[115]
**27N**	n.d.	0.24	0.85		7.6	0.71	[115]
**28N**	250	0.18	0.83		5.3	1.50	[78]
**29N**	250	0.18	0.80		5.3	0.63	[79]
**54N**	n.d.	0.20	1.2		5	0.70	[116]
**24N**	131	0.04	0.6		7	0.53	[65]
**25N**	131	0.04	0.3		7	0.98	[65]
**17N**	n.d.	0.05	0.19 (6.60)		4.5	0.36	[62]
**66N**	179	0.15	173% grafting		6	2.3	[117]
**30N**	250	0.18	0.23		5.6	1.07	[82]
**60N**	n.d.	0.15	0.75 (5.95)		5.5	0.45	[118]
**21N**	n.d.	0.17	n.d.	Ni(II)	6	1.00	[68]
**25N**	131	0.04	0.3		7	0.58	[65]
**30N**	250	0.18	0.23		5.6	1.12	[82]
**66N**	179	0.15	173% grafting		6	0.94	[117]
**30N**	2500	0.18	0.23	Co(II)	5.6	0.84	[82]
**25N**	131	0.04	0.3		7	2.03	[65]
**54N**	n.d.	0.20	1.2	Ag(I)	5	1.37	[116]
**28N**	250	0.18	0.83		5.3	1.53	[78]
**29N**	250	0.18	0.80		5.3	0.47	[79]
**65N**	n.d.	0.05	n.d.		4	0.79	[119]
**30N**	250	0.18	0.23		5.6	1.9	[82]
**61N**	n.d.	0.15	(6.33)		5.5	0.47	[120]
**62N**	n.d.	0.08	(10.62)		5.7	0.52	[121]
**56N**	50	0.20	n.d.	Hg(II)	7	2.09	[113]
**54N**	130	0.10	(4.56)		6	2.69	[114]
**17N**	n.d.	0.05	0.19 (6.60)		4.5	2.17	[62]
**19N**	n.d.	0.15	(4.01)		7	2.47	[69]
**18N**	n.d.	n.d.	n.d.		5	2.62	[70]
**25N**	131	0.04	0.3		7	2.82	[65]
**63N**	125	0.13	n.d.	Pd(II)	2	3.9	[87]
**58N**	n.d.	n.d.	(4.58)		2	1.03	[122]
**26N**	n.d.	0.05	0.9		0.01 M HCl	5.8	[75]
**28N**	250	0.18	1.0		0.1 M HCl	3.67	[78]
**28N**	250	0.18	0.83		0.1 M HCl	3.50	[80]
**29N**	250	0.18	0.80		0.1 M HCl	2.68	[79]
**65N**	n.d.	0.05	n.d.		4	2.04	[119]
**30N**	250	0.18	0.23		0.1 M HCl	2.35	[82]
**57N**	n.d.	n.d.	n.d.		2	1.13	[123]
**63N**	125	0.13	n.d.	Pt(IV)	2	2.0	[87]
**58N**	n.d.	n.d.	(4.58)		1	0.66	[122]
**28N**	250	0.18	1.0		0.1 M HCl	2.75	[78]
**28N**	250	0.16	0.83	Pt(IV)	0.1M HCl	2.6	[80]
**29N**	250	0.18	0.80		0.1 M HCl	1.61	[79]
**57N**	n.d.	n.d.	n.d.		2	0.63	[123]
**30N**	250	0.18	0.23		0.1 M HCl	1.9	[82]
**28N**	250	0.18	1.0	Au(III)	0.1 M HCl	5.56	[78]
**57N**	n.d.	n.d.	n.d.		2	0.86	[123]
**58N**	n.d.	n.d.	(4.58)		2	0.357	[122]
**30N**	250	0.18	0.23		0.1 M HCl	2.4	[82]
**16N**	371	0.05	0.6 (2.13)	Cr(VI)	6	0.61	[61]
**21N**	n.d.	0.17	n.d.	Zn(II)	6	0.9	[68]
**21N**	n.d.	0.17	n.d.	Cd(II)	6	0.9	[68]
**22N**	1000	0.20	0.5	As(V)	3	1.09	[63]
**22N**	1000	0.20	0.5	Se(IV)	3	0.81	[63]
**22N**	1000	0.20	0.5	Se(VI)	3	1.11	[63]
**23N**	n.d.	n.d.	n.d.	Mo(VI)	3-4	3.96	[66]

**Table 4:** Structure and sorption properties of S-containing chitosan derivatives

Chitosan Derivative	MW, kDа	Degree of Acetylation	S Content, mmol/g (Degree of Modification)	Sorption			Ref.
				Ion	pH	Capacity, mmol/g	
**67S**	125	0.13	0.53 (0.12)	Pd(II)	2	2.54	[30]
**68S**	125	0.13	0.81 (0.10)		2	3.24	[30]
**32S**	125	0.13	2.54 (0.54)		2	2.5	[87]
**39S**	250	0.18	0.93 (0.31)		2	1.24	[97]
**32S**	130	0.10	3.26		2	1.04	[89]
**40S**	n.d.	0	(0.90)		2	<0.3	[93]
**36S**	n.d.	0.05	(0.90)		2	5.8	[75]
**33S**	n.d.	0.05	(0.90)		2	4.8	[75]
**39S**	250	0.16	2.65 (0.63)		0.25 M HCl	2.91	[127]
**39S**	250	0.18	3.50 (0.90)		2	3.43	[96]
**44S**	250	0.18	1.37 (0.15)		2	2.47	[97]
**67S**	125	0.13	0.84 (0.18)	Pt(IV)	2	1.77	[100]
**32S**	125	0.13	2.54 (0.54)		2	2.0	[87]
**39S**	250	0.18	0.93 (0.31)		2	0.49	[97]
**39S**	250	0.18	3.50 (0.90)		2	1.24	[96]
**39S**	250	0.16	2.65 (0.63)		0.25 M HCl	1.41	[127]
**32S**	130	0.10	3.26		2	0.66	[89]
**36S**	n.d.	0.05	(0.90)		2	<0.3	[75]
**33S**	n.d.	0.05	(0.90)		2	<0.2	[75]
**44S**	250	0.18	1.37 (0.15)		2	1.12	[97]
**68S**	125	0.13	0.81 (0.10)	Au(III)	2	3.12	[31]
**70S**	125	0.13	n.d.		2	3.09	[31]
**39S**	250	0.18	3.50 (0.90)		2	3.81	[96]
**67S** magnetic	n.d.	n.d.	n.d.		0.5–2	3.6	[101]
**40S**	n.d.	0	(0.90)	Au(III)	2	<0.3	[93]
**36S**	n.d.	0.05	(0.90)		2	<0.3	[75]
**33S**	n.d.	0.05	(0.90)		2	<0.2	[75]
**32S**-Ag(I) templated magnetic	636	0.04	n.d.	Ag(I)	5	4.93	[88]
**40S**	n.d.	0	(0.90)		2	<0.4	[93]
**69S**	636	0.04	n.d.		4	3.77	[128]
**67S magnetic**	n.d.	n.d.	n.d.		6	2.1	[101]
**34S**	n.d.	0.07	2.54		6	0.54	[86]
**39S**	250	0.16	2.65 (0.63)	Hg(II)	5	3.04	[126]
**35S**	n.d.	0.05	6.60 (1.95)		4.5	0.82	[62]
**32S**	n.d.	0.05	2.54 (0.54)		4.5	2.93	[62]
**40S**	n.d.	0	(0.90)		2	<0.3	[93]
**34S**	n.d.	0.07	2.54		6	0.52	[86]
**32S**-Ag(I) templated	636	0.04	n.d.	Pb(II)	5	0.40	[88]
**69S**-Pb(II) templated	636	0.04	n.d.		6	2.02	[129]
**46S**	n.d.	0.22	2.66 (0.57)		4	2.24	[90]
**46S**	n.d.	0.22	2.66 (0.57)	Cu(II)	4	1.14	[90]
**32S**	n.d.	0.05	2.54 (0.54)		4.5	3.74	[62]
**35S**	n.d.	0.05	6.60 (1.95)		4.5	2.03	[62]
**40S**	n.d.	0	(0.90)		5	< 0.3	[93]
**45S**	n.d.	0.28	(0.22)		2	0.1	[91]
**34S**	n.d.	0.07	2.54		6	0.24	[86]
**43S**	n.d.	n.d.	n.d.		5.6	0.57	[95]
**31S**	n.d.	0.18	6.37 (1.94)		7	1.53	[84]
**47S**	400	0.26	n.d.		5	0.68	[92]
**42S**	n.d.	n.d.	n.d.	Co(II)	3.5	0.5	[94]
**31S**	n.d.	0.18	6.37 (1.94)		7	1.13	[84]
**40S**	n.d.	0	(0.90)	Ni(II)	5	< 0.3	[93]
**31S**	n.d.	0.18	6.37 (1.94)		7	1.25	[84]
**46S**	n.d.	0.22	2.66 (0.57)	Cd(II)	4	1.14	[90]
**31S**	n.d.	0.18	6.37 (1.94)	Zn(II)	7	0.83	[84]
**42S**	n.d.	n.d.	n.d.	Eu(III)	8	0.23	[94]

**Table 5:** Structure and sorption properties of P-containing chitosan derivatives.

Chitosan Derivative	MW, kDа	Degree of Acetylation	Degree of Modification	Sorption			Ref.
				Ion	pH	Capacity, mmol/g	
**48P**	n.d.	0.03	1.3	Cu(II)	7.4	2.6	[103]
				Ni(II)		2.3	
				Zn(II)		2.7	
				Sr(II)		1.7	
				Ca(II)		1.5	
				Mg(II)		1.3	
				Cd(II)		3.0	
**49P**	50	0.8	1.57	Cu(II)	7	1.51	[105]
				Ca(II)		1.55	
				Mg(II)		1.46	
				Cd(II)		1.65	
**48P**	n.d.	n.d.	n.d.	U(IV)	n.d.	0.069 × 10^−3^	[104]

**Table 6:** Stability constants for complexes of transition metals with chitosan and its derivatives at the metal:ligand ratios 1:1 (ML) and 1:2 (ML_2_).

Ion	Complex Composition	Chitosan	*N*,*O*-Carboxymethyl Chitosan *	*N*-(4-hydroxysalicylidene) Chitosan
Cu(II)	ML ML_2_	5.47 [142] 8.09 [142]	10.54 [14]	5.25 [151]
Ni(II)	ML ML_2_	3.80 [148] 7.01 [148]	8.32 [14]	4.71 [151]
Co(II)	ML ML_2_	4.18 [149] 8.29 [149]	6.34 [14]	5.72 [151]
Zn(II)	ML ML_2_	4.03 [149] 6.47 [149]	7.24 [14]	5.73 [151]
Mn(II)	ML ML_2_	3.75 [148] 6.91 [148]	5.30 [14]	-

* stability constants of *N*-(2-carboxy)ethyl chitosan complexes with degrees of substitution 0.42 (CuL_2_), 0.92 (CuL_2_), and 1.91 (CuL) were equal to 10.06, 11.06, and 6.41, respectively [46].
